# Structural and mutational analysis reveals that CTNNBL1 binds NLSs in a manner distinct from that of its closest armadillo-relative, karyopherin α^☆^

**DOI:** 10.1016/j.febslet.2013.11.013

**Published:** 2014-01-03

**Authors:** Karuna Ganesh, Febe van Maldegem, Stephanie B. Telerman, Paul Simpson, Christopher M. Johnson, Roger L. Williams, Michael S. Neuberger, Cristina Rada

**Affiliations:** Medical Research Council Laboratory of Molecular Biology, Francis Crick Avenue, Cambridge CB2 0QH, UK

**Keywords:** Splicing, Nuclear import, Armadillo domain

## Abstract

CTNNBL1 is a spliceosome-associated protein that binds nuclear localization signals (NLSs) in splice factors CDC5L and Prp31 as well as the antibody diversifying enzyme AID. Here, crystal structures of human CTNNBL1 reveal a distinct structure from its closest homologue karyopherin-α. CTNNBL1 comprises a HEAT-like domain (including a nuclear export signal), a central armadillo domain, and a coiled-coil C-terminal domain. Structure-guided mutations of the region homologous to the karyopherin-α NLS-binding site fail to disrupt CTNNBL1–NLS interactions. Our results identify CTNNBL1 as a unique selective NLS-binding protein with striking differences from karyopherin-αs.

## Highlights

• The structure of CTNNBL1 includes an abbreviated armadillo domain.
• CTNNBL1 is a novel NLS binding protein with a unique carboxy-terminal structure.
• Structure-based mutagenesis of CTNNBL1 shows NLS binding distinct from karyopherins.

## Structured summary of protein interactions

**CTNNBL1**binds to **CDC5L NLS3** by pull down (View interaction)

**CTNNBL1** and **CDC5L NLS3**bind by isothermal titration calorimetry (View interaction)

**CTNNBL1** and **Prp31 NLS**bind by isothermal titration calorimetry (View interaction)

## 1 Introduction

CTNNBL1 is a widely expressed, highly conserved 62 kDa nuclear protein [1] that associates with components of the RNA splicing machinery [2], [3], [4]. Mice lacking CTNNBL1 die in mid-gestation, whilst CTNNBL1-deficient mouse primary B cells and yeast show delayed exit from quiescence [5]. Whilst CTNNBL1 lacks significant sequence similarity to other proteins, secondary structural predictions suggest that CTNNBL1’s central region might comprise an armadillo domain, a multifunctional domain that is found in several unrelated proteins [1], [6]. CTNNBL1 has been predicted to resemble the karyopherin-α nuclear transport adaptors, in which the armadillo domain surface groove mediates interactions with nuclear localization signals (NLSs) in cargo proteins. In previous work we have shown that like the karyopherin-αs, CTNNBL1 binds to NLSs with high selectivity and affinity, in particular the NLS region of essential spliceosome components CDC5L and PRPF31, as well as the ill defined NLS of the antibody diversification enzyme AID [4]. However, CTNNBL1 has an NLS binding specificity distinct from that of karyopherin-α, failing, for example, to bind to the NLS of the SV40 T-antigen [4]. To gain insight into the distinct NLS binding preference of CTNNBL1, we solved the crystal structure of CTNNBL1, and based on the structure, probed the putative NLS binding region by mutagenesis. The results show a distinctive alpha helical protein, only partially comprised of armadillo repeats, with striking dissimilarities from karyopherin-α.

## 2 Methods

### 2.1 Protein expression, purification and crystallization

CTNNBL1 and CTNNBL1Δ76 were expressed and purified as described previously [4]. CTNNBL1Δ76 mutants were generated by PCR using the primers shown in Supplementary Table 1. Selenomethionine-labeled protein was expressed in B834(DE3) cells as described previously [7] and purified as for unlabeled protein.

Purified recombinant proteins at a concentration of 24 mg ml^−1^ in crystallization buffer (20 mM Hepes pH 7.5, 50 mM NaCl, 1 M DTT) were tested in crystallization screens with 1536 different conditions in sitting drops (100 nl protein solution + 100 nl reservoir solution) in 96-well MRC crystallization plates using a Cartesian robot (Genomics solutions, Huntingdon, UK). Optimal crystals were grown in sitting drops in 24-well crystallization plates (CrysChem) by mixing 0.5 ml protein solution with 0.5 ml reservoir solution. Solutions and cryoprotection details are listed in Supplementary Table 2.

### 2.2 Data collection

Data were collected at 100 K from cryoprotected crystals frozen in a nitrogen gas stream. MAD datasets from the selenomethionine crystal, CTNNBL1 and CTNNBL1Δ76B (crystal form B of CTNNBL1Δ76) were collected at the Diamond Light Source beamline I02, while CTNNBL1Δ76A (crystal form A of CTNNBL1Δ76) was collected at beamline I04-1. Details of the methods used for Phasing and Model Refinement are described in Supplementary methods. Crystal statistics are given in Table 1.

**Table 1:** Data collection, phasing and refinement statistics.

Crystal name	CTNNBL1Δ76A	CTNNBL1Δ76B	CTNNBL1	SeMetCTNNBL1		
Space group	*P*2_1_ 2_1_ 2_1_	*I*2 2 2	*P*4_3_ 2_1_ 2		*P*4_3_ 2_1_ 2	

*Cell dimensions*						
*a*, *b*, *c* (Å)	63.1, 92.7, 121.7	87.4, 92.8, 194.9	66.7, 66.7, 325.9		66.8, 66.8, 328.2	
				*Peak*	*Inflection*	*Remote*
Wavelength (Å)	0.979500	0.979500	0.979500	0.979700	0.97980	0.96820
Resolution (Å)	63–2.9 (3.08–2.9)	40–3.1 (3.3–3.1)	81–3.0 (3.2–3.0)	47.36–2.32	47.36–2.32	47.46–2.32
*R*_merge_	6.8 (49.1)	9.7 (39.8)	11.2 (91.6)	21 (−165)	21.7 (238.7)	2.2 (395.4)
*I*/*σI*	14.6 (3.3)	10.5 (3.6)	16.4 (3.1)	7.3 (−0.1)	7.4 (0.6)	7.7 (0.8)
Completeness (%)	97.6 (99.0)	98.8 (98.4)	99.96 (100)	64.6	62.4	62.2
Redundancy	4.0 (4.1)	5.0 (5.1)	13.4 (13.6)	6.2	6.3	6.3

*Refinement*						
Resolution (Å)	52.1–2.9	39.9–3.1	65.4–3.0		47.42–3.3	
No. of reflections	15031	13833	14963			
*R*_work_/*R*_free_	19.2/26.9	21.7/27.9	21.8/29.8			
No. atoms						
Protein	3924	3968	4023			
${\text{SO}}_{4}^{3-}$	2	4	0			
Water	5	8	2			
*B*-Factors	67.9	69.17	90.886			
R.m.s deviations						
Bond lengths (Å)	0.011	0.011	0.009			
Bond angles (°)	1.43	1.74	1.35			

One crystal was used per data set. Values in parentheses are for the highest resolution shell.

### 2.3 Assaying peptide binding

Peptides corresponding to the CDC5L NLS3, SV40 NLS and Prp31 NLS and CTNNBL1Δ76 mutants were dialysed against isothermal titration calorimetry (ITC) buffer (20 mM Hepes pH 7.5, 50 mM NaCl) and concentrated to 0.83 mM (peptide) or 50 μM (protein) for experiments on an ITC_200_ or auto-ITC_200_ calorimeter (Microcal, Inc.). ITC and pulldown assays were performed as described previously [4].

### 2.4 Protein localization

293T cells transiently transfected with N-terminally GFP-tagged CTNNBL1 fragments were fixed, permeabilized, counterstained with Hoechst 3258 and Alexa-594 conjugated wheatgerm agglutinin, and visualized using confocal microscopy as described [4].

## 3 Results and discussion

### 3.1 Crystal structures of human CTNNBL1

We crystallized full-length human 6xHis-CTNNBL1 and N-terminally truncated 6xHis-CTNNBL1Δ76 (to generate a fragment that is necessary and sufficient for NLS-peptide binding [4]). We were unable to solve the structure using molecular replacement and therefore crystallized selenomethionine-substituted protein for experimental phase determination, solving the structures of CTNNBL1Δ76 and that of full-length CTNNBL1 from crystals with the symmetry of different spacegroups (Table 1).

**Fig. 1:**
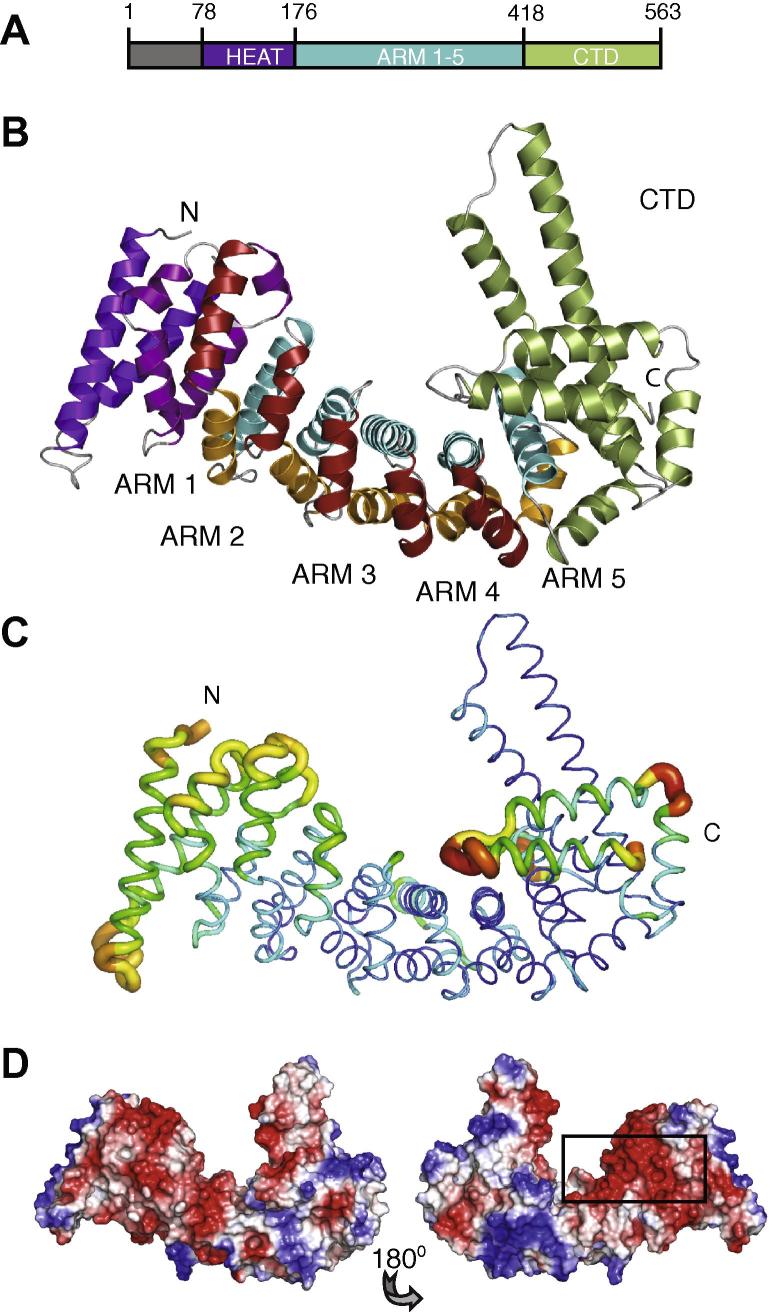
Structure of CTNNBL1. (A) Architecture of CTNNBL1 with the N-terminal unresolved region, HEAT-like domain, five armadillo repeats (ARM) and C-terminal domain (CTD). (B) Three-dimensional structure of CTNNBL1. The PRE-ARM domain HEAT-like repeats are in pink and purple; ARM repeats indicated with helix 1, helix 2 and helix 3 in red, orange and cyan and the CTD is shown in green. (C) B-factors representation in CTNNBL1 crystals. Lower B-factors are blue and are thinner while thicker regions in yellow to red have higher B-factors. (D) Electrostatic surface potential of CTNNBL1. (Red, negative; blue, positive). Views rotated by 180°. Acidic groove boxed.

The crystals reveal the same overall structure of CTNNBL1. However, despite excellent continuity within the protein chain, we were unable to resolve any density for the N-terminal (76) amino acids in the structure of full length CTNNBL1. The overall structure of CTNNBL1Δ76 reveals a superhelical coiled coil composed of α-helices stacked against each other to form an extended configuration, in agreement with circular dichroism data (Supplementary Fig. 2B). The structure can be divided into three distinct regions (Fig. 1A): an N-terminal pre-armadillo (PRE-ARM) domain (residues 76–176); a central compact armadillo (ARM) domain with helices tightly packed against one another to form a solenoid (residues 177–416); and a C-terminal domain (CTD) that projects out from the smooth solenoid of the ARM domain (residues 417–562) (Fig. 1B).

Residues along the central spine of the solenoid and the CTD have low temperature factors, consistent with their role in stabilizing the core of the molecule, whilst N and C-terminal regions beyond the CTD display higher B-factors for solvent-exposed residues, implying greater flexibility (Fig. 1C). The solenoid core forms a concave electronegative groove, comprised primarily of acidic amino acids from helices 1 and 3 of the armadillo domain, similar to the acidic concave groove of karyopherin-α [8] and unlike the positively charged groove formed by β-catenin [6], [9], [10] (Fig. 1D). In contrast to the negatively charged core, patches of positive charge are located on the periphery.

### 3.2 The pre-armadillo domain of CTNNBL1 contains a HEAT repeat-like region

**Fig. 2:**
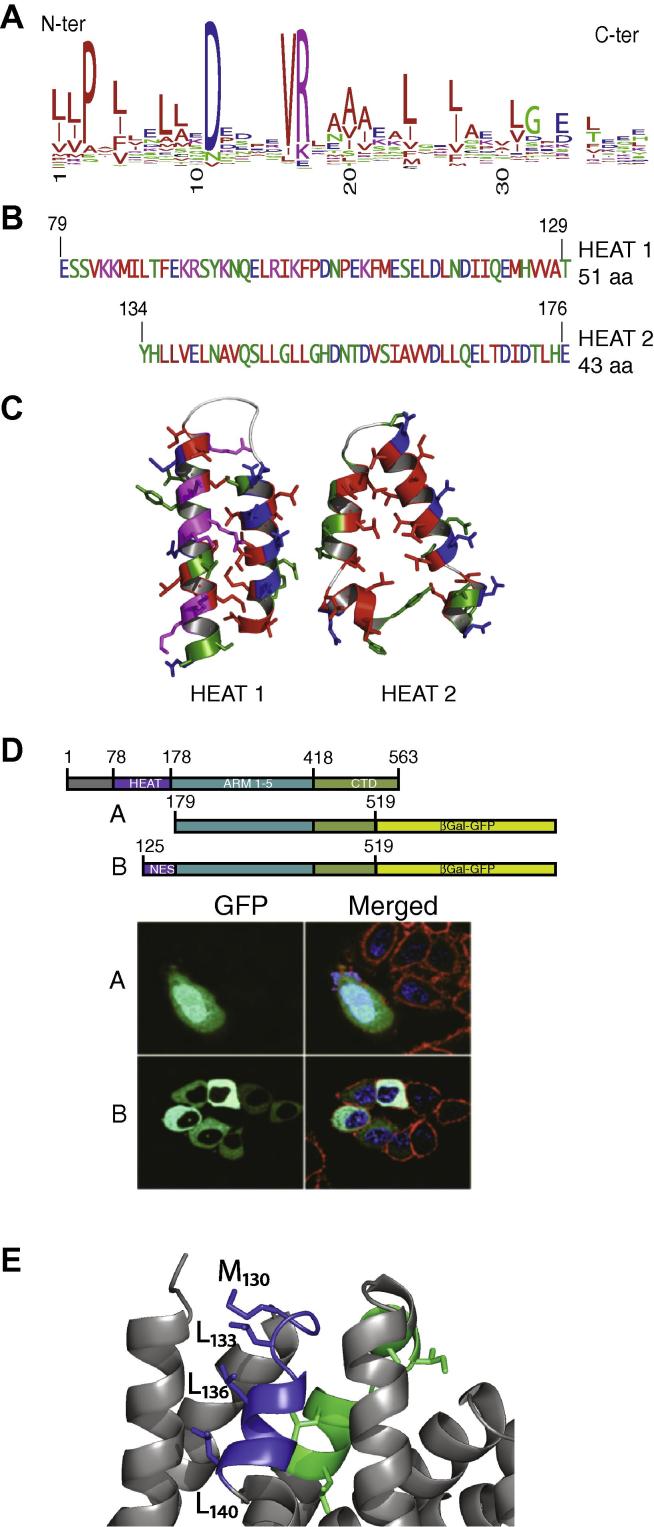
The PRE-ARM domain of CTNNBL1 contains HEAT-like motifs comprising a nuclear export signal. (A) Consensus HEAT repeat (as in Supplementary Fig. 4) (Residues are colored acidic, blue; basic, pink; hydrophobic, red and non-polar, green). (B) Amino acid sequence of HEAT-like regions 1 and 2 of CTNNBL1. (C) The structures of the HEAT-like regions are stabilized by conserved hydrophobic residues (colored as in A). (D) The PRE-ARM domain CTNNBL1 contains a functional export signal. Cytoplasmic relocalization of a chimera of CTNNBL1 residues 179–519-β-galactosidase-GFP in transfected 293T cells by addition of residues 125–179. (E) Detail shows in blue (residues 130–140), the putative functional NES region with the key leucine residues depicted as sticks. The predicted NES in Jabbour et al. is shown in green indicating the leucines buried inside the core of the protein.

Whilst showing no amino acid sequence homology to other armadillo containing proteins, secondary structure predictions have suggested that CTNNBL1 comprises multiple armadillo repeats although the number and extent of these repeats was difficult to define [1]. The crystal structure shows that the armadillo domain of CTNNBL1 begins at E178. Preceding the ARM domain is a region (PRE-ARM domain) comprising 6 α-helices and connecting loops, which do not form the ordered triple helical arrays that constitute armadillo repeats [9], [11], [12], [13]. Notably, this region lacks the highly conserved glycines at the junction between the first and second helices that are a hallmark of armadillo repeats [13]. Instead, residues Q79 to T129 form a pair of antiparallel helices with the remaining four helices of the PRE-ARM domain (residues Y134 to E178) resembling an intermediate type of motif that falls between that of a modified armadillo repeat (with the insertion of an extra C-terminal helix) and a HEAT-like repeat (with two kinked helices A and B) (Fig. 2A–C). The HEAT repeat, which is a relative of the armadillo repeat, consists of a tandem array of two antiparallel helices (A and B) instead of the three that comprise the armadillo repeat, although helix A often contains a central proline residue that leads to kinking of this helix [13], [14], [15].

While attempts to narrowly define the HEAT domain have been made (e.g. [13], [16]), recent accumulation of structural data has shown that HEAT repeats are highly variable in length, position of hydrophobic residues and the conservation of other residues (such as P3, D11 and R18) (Fig. 2A and Ref. [12]). As in HEAT repeats, the CTNNBL1Δ76 PRE-ARM helices also contain conserved hydrophobic residues in the buried interaction surface of the helices where they mediate intra- and inter-repeat packing. Polar residues line the solvent-exposed faces of the amphipathic helices (Fig. 2B and C), and conserved prolines kink the α-helices. Another feature of HEAT repeats is the presence of conserved polar residues near the surface inter-helix loop, which mediate contact between the two helices [13]. In CTNNBL1, N96 interacts with S113 and H152, and R100 interacts with E114. Thus the pre-ARM domain of CTNNBL1 is an α-helical region that transitions between a HEAT and an armadillo repeat topology, explaining the difficulties in predicting the precise boundaries of the ARM core domain.

### 3.3 The HEAT-like region of CTNNBL1 contains a functional nuclear export sequence

While CTNNBL1 is actively localized to the nucleus [1], [4], Jabbour et al. suggested that residues 164–174 (LLQELTDIDTL) could constitute a nuclear export sequence (NES) [1]. The structure reveals that these residues are unlikely to have this role since the leucine residues are buried in the protein core. Nonetheless, addition of residues 125–179 to the armadillo domain of CTNNBL1 causes complete cytoplasmic relocalization of a GFP-fusion protein, while the armadillo domain on its own remains entirely nuclear, confirming that the region contains an active NES (Fig. 2D). Leucine-rich NESs are typically located in mobile regions of proteins, close to the surface, at a structural transition between a stacked amphipathic helix and an extended loop [17], [18]. On inspection of the structure, there is only one part of the CTNNBL1 PRE-ARM domain that meets these criteria: residues 130–140 (MPDLYHLLVEL) have high B factors, and form a transition from a loop to a helix protruding outwards at the surface of CTNNBL1 (Fig. 2E). The hydrophobic side chains of residues M130, L133, L136, L140 are exposed on one side of an amphipathic helix, with D132, Y134, E139 on the opposite face. Based on the crystal structure, CTNNBL1 residues 130–140 could therefore constitute an active nuclear export sequence.

### 3.4 The C-terminal domain of CTNNBL1 constitutes a novel protein fold containing a coil–coil

**Fig. 3:**
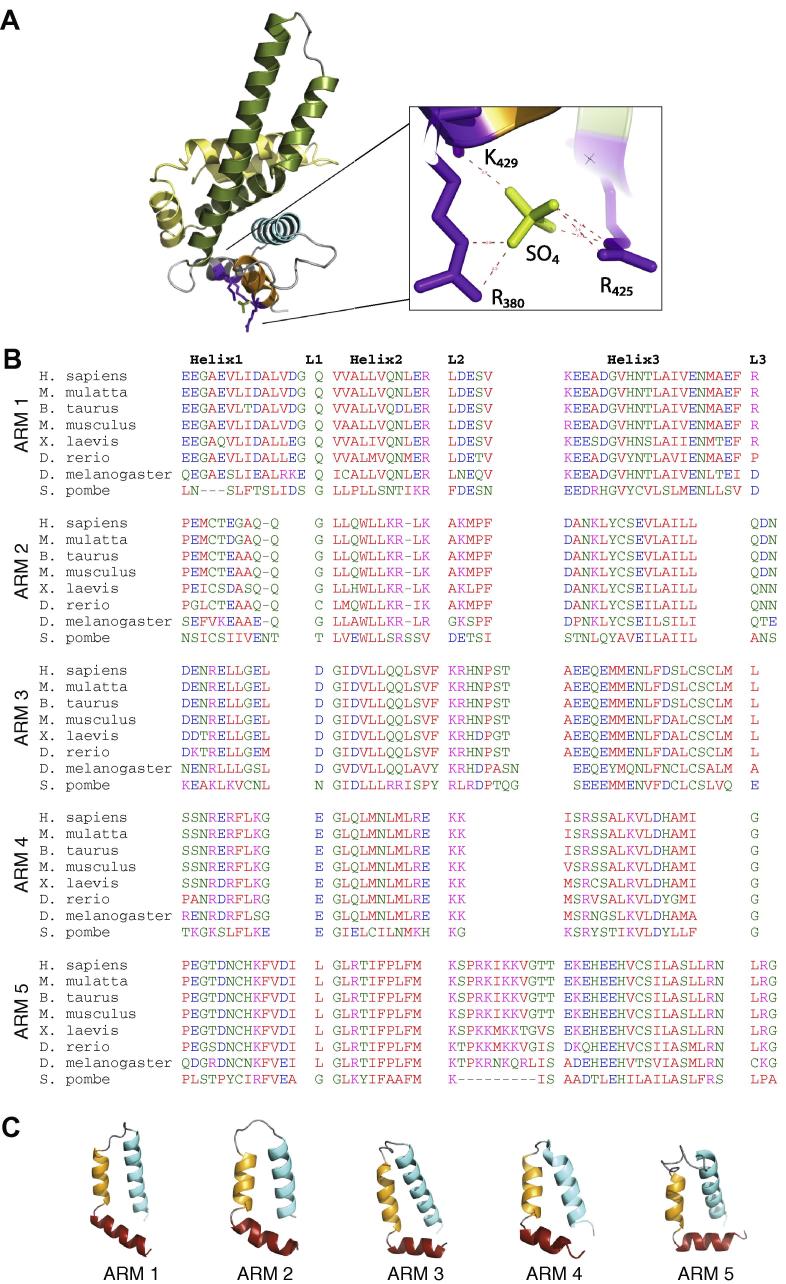
The rigid CTD of CTNNBL1 terminates the conserved ARM domain of CTNNBL1. (A) Diagram showing helices 2 and 3 of armadillo repeat 5 (orange and cyan) and the CTD coiled-coil (green) and wing-like region (yellow). Hydrophobic residues stabilizing the base of the CTD are shown in purple. (B) Structure-based multiple sequence alignment of the 5 armadillo repeats of CTNNBL1, including *S. pombe*. (C) Structural variation among individual armadillo repeats of CTNNBL1. Repeats are oriented vertically along the central axis.

The most dramatic feature of the structure is the abrupt termination of the HEAT/ARM solenoid by a rigid extended 34 aa helix and an antiparallel pair of helices 14 and 17 aa long, separated by G489. Despite the presence of the glycine residue, the whole region is quite rigid (as suggested by the B-factors (Fig. 1C), although the 108° angle between the latter two helices forms a kink that makes the coiled-coil region wider at the distal end from the axis of the solenoid. The rigidity of the structure is achieved by a dense cluster of hydrophobic interactions among the last arm motif H3 helix and CTD helices 1, 2, 4 and 5, forming a tightly packed base for the CTD (Fig. 3A). Although residues L519, I526, I531 and I540 are arranged in the heptad pattern typical of leu-ile zipper motifs, the tertiary structure shows that the side-chains point towards the protein core and do not participate in the typical interactions of a Leu-Ile zipper [19], in contrast to prior prediction [1]. The wider distal part of the coiled-coil is less conserved (Supplementary Fig. 1) although an acidic patch is still present in fission yeast *Schizosaccharomyces* *pombe* (residues D462, E464, E465 in *S. pombe* and D476, E479, E480 in human). The last three helices of the CTD form an additional flexible armadillo-like repeat.

The base of CTD is abutted perpendicularly to the main axis of the solenoid, with a density corresponding to a sulfate ion buried deep within a highly conserved pocket formed by R380, R425 and K429, all making polar contacts. It is possible that the sulfate in the crystals mimic a natural (e.g. phosphorylated) ligand and that this interaction contributes to the stability of the coil-coil region, modulating the position of the CTD and access to the concave surface of the solenoid.

### 3.5 The core of CTNNBL1 comprises a compact array of five armadillo repeats

Although Jabbour et al. had predicted that CTNNBL1 might contain a large ARM domain comprising 10 armadillo repeats [1], analysis of the crystal structure reveals a much smaller ARM domain, with only five armadillo repeats similar to those found in other ARM proteins [12], [13] (Fig. 1A). The repeats range in size between 36 and 45 amino acids, and measure about 30 Å in diameter (Fig. 3B). Each armadillo repeat is organized into three alpha helices, with helix 1 arranged perpendicularly to helices 2 and 3, which in turn run antiparallel to one another (Fig. 3C). As in consensus ARM repeats, the helix 1s are short, and helix 3s are longest (up to 20 amino acids) and most highly conserved. The long helix 3s stack against one another to form a concave groove, with an average translational distance between adjacent repeats of 7.9 Å.

The individual ARM repeats in the CTNNBL1 structure conform to the overall ARM consensus. They show considerable sequence diversity from repeat to repeat, but the overall array is highly conserved among CTNNBL1 homologues (Fig. 3B and C, Supplementary Fig. 2). Each repeat contains at least one conserved glycine at the junction between helices 1 and 2, a hallmark of ARM motifs, with backbone dihedral angles in the α_L_ region of the Ramachandran plot that permit the sharp 90° bend between helices 1 and 2 [13]. The loops between helices 2 and 3 are more variable in length, have higher B factors and have the ability to form polar contacts with neighbouring loops (Fig. 3C). With the exception of ARM 5 in lower eukaryotes, the size of both helices and intervening loops is well conserved.

### 3.6 The concave surface of the CTNNBL1 core ARM domain is not essential for NLS binding

**Fig. 4:**
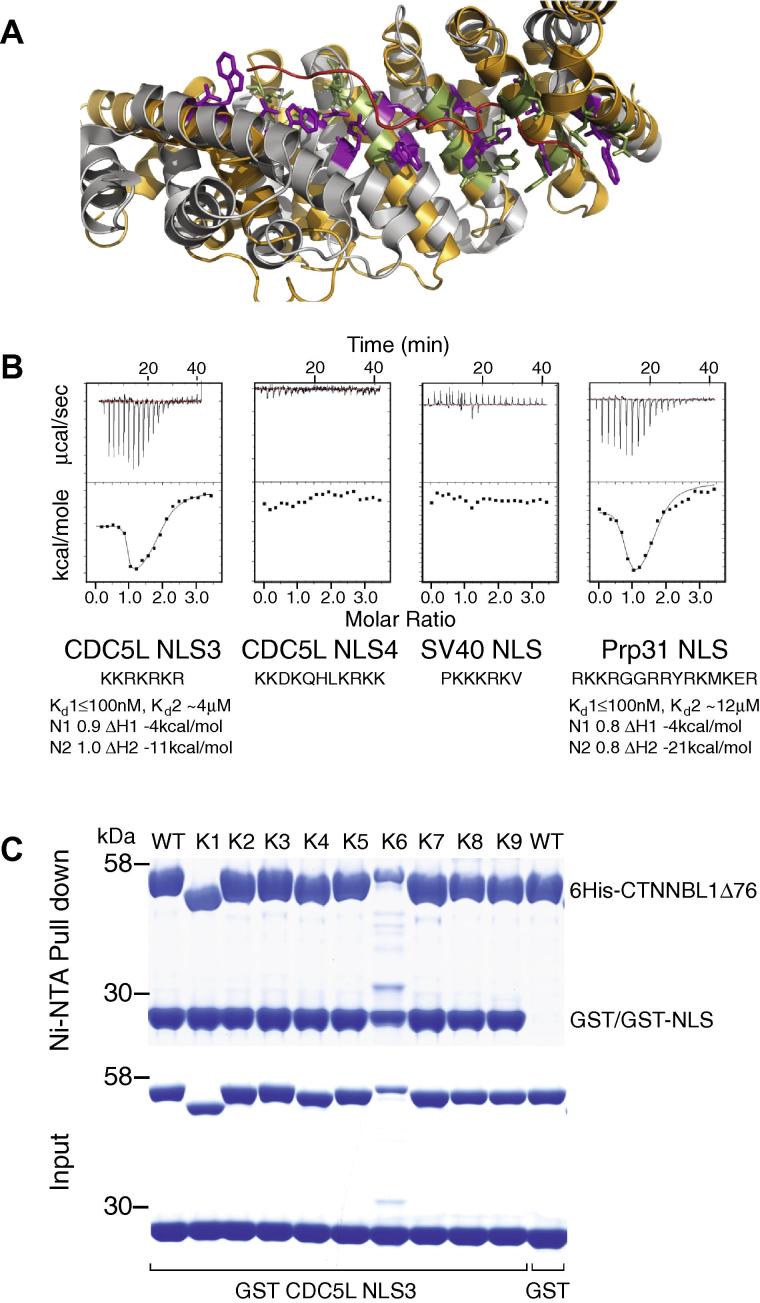
CTNNBL1 binding to NLS peptides. (A) Overlay of mouse karyopherin-α (grey) bound to the NLS of Nucleoplasmin (red) (PDB file1EJY, [21]) superimposed on the structure of CTNNBL1 (orange). Conserved tryptophans and arginines in karyopherin-α that mediate NLS binding are shown in purple with equivalent residues in CTNNBL1 in green (residues 519–563 and part of the CTD are excluded for clarity). (B) Selective binding of CTNNBL1Δ76 to basic peptides of CDC5L–NLS3 and Prp31 compared to CDC5L–NLS4 and SV40 T-antigen measured by ITC. Sequences corresponding to the NLSs peptides are shown. (C) 6His-CTNNBL1Δ76 and mutants (as in Table 2) interact with GST-CDC5L-NLS3 fusion proteins but not GST alone. Bound proteins were visualized with Coomassie blue following SDS–PAGE.

The compact core of CTNNBL1 ARM domain creates a concave groove similar to karyopherin-α with a linear surface patch of negative charge [8] (Figs. 4A and 1D). In karyopherin-αs, both the N-terminal [2], [3], [4] and C-terminal [7], [8] ARM repeats are directly involved in binding to bipartite NLSs like nucleoplasmin, whereas the major N-terminal site alone is used in binding the monopartite SV40 large T antigen or Myc NLSs [8], [20], [21]. Like karyopherin-αs, CTNNBL1 also shows selective binding to positively-charged NLSs, exhibiting strong binding to the Prp31 NLS or NLS3 of CDC5L but with no binding to the SV40 NLS or to NLS4 of CDC5L (Fig. 4B). The specific interaction observed by ITC produce complex binding isotherms best described using a two independent site model with *K*_d_’s of ∼0.1 and 5 μM, as we have previously reported [4].

In order to probe the NLS binding mode of CTNNBL1, we generated multiple mutants of CTNNBL1Δ76 based on the crystal structure (Table 2 and Fig. 4A). Deletion of the C-terminal wing-like region of CTNNBL1, [Δ521-563], did not affect NLS binding (Fig. 4C) nor the stability or overall folding of the protein as determined by Circular dichroism (CD) spectroscopy (not shown). Multiple mutants of CTNNBL1 were also generated that carried amino acid substitutions in the putative NLS recognition groove (Fig. 4A). Strikingly, all mutants retained the ability to interact with the CDC5L NLS3 as judged by a pulldown assay (Fig. 4C). Indeed, not only was NLS binding retained by these mutants, but the affinity of binding to the CDC5L NLS3 (as well as to the Prp31 NLS) peptide, as monitored by ITC, was little affected by these amino acid changes; the small differences in binding affinity, such as the deletion mutant K1, might reflect a minor influence on the stability of the protein (Supplementary Fig. 5). Thus, CTNNBL1 likely recognizes NLS ligands in a manner very different from karyopherin-α.

**Table 2:** Description of CTNNBL1Δ76 mutants.

Mutation^a^	Location
WT	CTNNBL1Δ76
K2: D153 N154 T155 D156	Pre-ARM
K4: D170 D172	Pre-ARM^b^
K6: Q166	Pre-ARM
K5: H216N217	ARM1 Helix3
K7: E223N224 Y261	ARM1 & ARM2 Helix3
K8: E264I268	ARM2 Helix3
K9: D315S319H359	ARM3 & ARM4 Helix3
K3: L321 M322H359 I362	ARM3 & ARM4 Helix3
	
Deletion [Δ521–563]	CTD

Underlined are residues structurally equivalent to the NLS binding WxxxN motif of karyopherin-α as depicted in Fig. 4A.

a Substitutions to alanine.

b Residues affecting the acidic pocket in helix 5 of the Pre-ARM.

## 4 Conclusions

CTNNBL1 has a highly compact ARM domain with a novel and rigid C-terminal coiled-coil domain. The N-terminus contains a disordered region and a structured HEAT-like region that includes a functional NES. Despite structural similarities with karyopherin-α, our data suggest a very different NLS binding mode that does not seem to rely on the concave groove formed by the ARM domain. These functional data are in agreement with the lack of conservation of the key tryptophan and asparagine residues that line the groove formed by the ARM helix 3s of karyopherin-α (WxxxN motif, see Fig. 4A and Fig. S4) [8], [20], [21] which mediate NLS binding through hydrophobic interactions to the basic side chains and hydrogen bonding to the main chains of the peptide. The lack of amino acid sequence conservation between karyopherin-α and CTNNBL1 therefore reflects a distinct NLS binding mode and may account for the selectivity of the NLS interactions of CTNNBL1.

The abbreviated ARM domain of CTNNBL1 also precludes space for a potential minor groove as in karyopherins. However, the presence of other patches of negative electrostatic potential, including the extended C terminal helix coil-coil region or the acidic patch between the HEAT-like region and the core ARM domain, opens the possibility of multiple binding sites for NLS containing proteins (such as the NLS region of CDC5L). Indeed, a model docking of one of the interacting partners of CTNNBL1, AID, into the independently determined structure of CTNNBL1 (reported by Huang et al. while this manuscript was in preparation [22]), suggests that the CTD region might be involved in binding to NLSs. While their model invokes D315, E264 and E223 for the interaction with AID these residues are not required for binding to the spliceosome NLSs. Further functional, structural and biochemical analysis should clarify the distinctive nature of the binding of CTNNBL1 to its NLS-containing partners.

## PDB references

CTNNBL1Δ76A 4cb8, CTNNBL1Δ76B 4cba and CTNNBL1 4cb9.

## Appendix A Supplementary data

Supplementary data 1 This document contains Supplementary tables.

Supplementary Fig. 1A

Supplementary Fig. 1B
