# Supplementary material for: Structural and mutational analysis reveals that CTNNBL1 binds NLSs in a manner distinct from that of its closest armadillo-relative, karyopherin α
Source: FEBS Lett. 2014 Jan 3;588(1):21–7. doi: 10.1016/j.febslet.2013.11.013 (PMC3885797; doi:10.1016/j.febslet.2013.11.013)
Supplement: Supplementary data 1 — This document contains Supplementary tables. [file mmc1.docx]

**Supplementary data**

Supplementary Table 1. Primers^a^ used to generate the CTNNBL1Δ76 and mutant recombinant proteins

6His-CTNNBL1Δ76 in pOPTH: forward primer (spanning pOPTH Multicloning XbaI site): GGCTCCGGAGAGCTCCAATTGG and reverse with XbaI cloning site: GCCTCTAGAAGTTCTCCAGCAAGCCCAGGATGC

K1 ctggtctagactaCATGTTTAGGATCTGGTGAACC

K2 GGCTTGCTCGGACACGcTgcTgCAGcTGTGTCCATAGCTGTGG

CCACAGCTATGGACACAgCTGcAgcAgCGTGTCCGAGCAAGCC

K3 CCTGAAAGTGCTGGACgcTGCCATGgcTGGCCCCGAAGGCACAG

CTGTGCCTTCGGGGCCAgcCATGGCAgcGTCCAGCACTTTCAGG

GATTCCCTCTGCTCCTGTgcAgcGCTTAGTTCCAATCGTG

CACGATTGGAACTAAGCgcTgcACAGGAGCAGAGGGAATC

K4 CAGGAATTAACAGcTATAGcCACCCTCCATGAGAGTGAAG

CTTCACTCTCATGGAGGGTGgCTATAgCTGTTAATTCCTG

K5 GGAGGCAGATGGCGTCgcCgcCACTCTGGCTATTGTGG

CCACAATAGCCAGAGTGgcGgcGACGCCATCTGCCTCC

K6 GTGGTCGATTTGCTTCcGGAATTAACAGATATAGAC

GTCTATATCTGTTAATTCCgGAAGCAAATCGACCAC

K7 CACTCTGGCTATTGTGGcAgcCATGGCTGAGTTCCG

CGGAACTCAGCCATGgcTgCCACAATAGCCAGAGTG

CCTTTTGATGCCAACAAACTGgcTTGCAGTGAAGTGCTGG

CCAGCACTTCACTGCAAgcCAGTTTGTTGGCATCAAAAGG

K8 CAAACTGTATTGCAGTGcAGTGCTGGCCgcATTGCTCCAGGACAATG

CATTGTCCTGGAGCAATgcGGCCAGCACTgCACTGCAATACAGTTTG

K9 GATGGAGAATCTGTTTGcTTCCCTCTGCgCCTGTCTAATGCTTAG

CTAAGCATTAGACAGGcGCAGAGGGAAgCAAACAGATTCTCCATC

CCTGAAAGTGCTGGACgcTGCCATGATTGGCCCC

GGGGCCAATCATGGCAgcGTCCAGCACTTTCAGG

^a^ Modified nucleotides are non-capitals

Supplementary Table 2. Composition of the crystallization reservoir solutions and cryoprotection methods

|  | Reservoir Solution | Cryoprotection Method |
| --- | --- | --- |
| CTNNBL1Δ76A | 26% PEG 3350, 0.2 M Li2SO4, 0.1 MTris-Hcl pH 8.5 | serial equilibration with mother liquor solution containing 5% then 25% glycerol |
| CTNNBL1Δ76B | 0.12 M ethylene glycol, 12.5% PEG 3350, 12.5% PEG 1K, 12.5% MPD, 0.1 M Bicine/Trizma base | serial equilibration with mother liquor solution containing 5% then 25% glycerol |
| CTNNBL1 | 0.1 M Tris pH 8.5, 1.1 M NaF, |  |
| 20% PEG 8K, 4.5% PEG 550 MME, 0.1 M TCEP hydrochloride | serial equilibration with mother liquor solution containing 1% then 5% then 25% MPD |  |
| SeMetCTNNBL1 | 0.1 M Tris HoAc pH 8.5, 18% PEG 8K, |  |
| 7.6% PEG 550 MME, 1.6 M NaF | serial equilibration with mother iquor solution containing 1% then 5% then 25% MPD |  |

**Supplementary methods**

Phasing and Model Refinement details

Data for SeMet CTNNBL1 were processed using CCP4i (1); integrated using Xia2 (2) and scaled using SCALA (3). 7 out of 7 possible selenium atoms in the asymmetric unit were located using SHARP (4) and refined using AUTOSHARP (5). Solvent flattening was performed using Parrot. An initial model was built using Buccaneer (6), and manually adjusted in COOT (7). The structure was refined using RefMac5 (8). Data for the selenomethionine was used to find phases for the native crystal data via molecular replacement using Phaser (9); model building and refinement were as above.

ITC

The complex binding isotherms of the CTNNBL1/NLS peptide interaction could be best described using the two independent site model in the ITC Origin software with Kd’s of ~ 0.1 and ~5 μM (as in reference 4 main text). Errors on these values are significantly higher than would be expected from simpler ITC titrations both because of the high affinity of the first Kd, which is at the limit for the ITC technique, and due to the reduction in data density as points are split between the two binding events. The absence of any apparent ITC signal from the SV40 NLS or CDC5L NLS4 peptides indicates that both components of the complex ITC data report on specific binding events. Such a model is supported by ITC data on binding of SV40 NLS to karyopherin-α where two binding sites were reported with Kd’s of 0.3 and 1 μM (10).

**Supplementary References**

1. Potterton E, Briggs P, Turkenburg M, Dodson E. A graphical user interface to the CCP4 program suite. Acta Crystallogr D Biol Crystallogr. 2003 Jul;59(Pt 7):1131-7.
2. Winter G, Lobley CM, Prince SM. Decision making in xia2. Acta Crystallogr D Biol Crystallogr. 2013 Jul;69(Pt 7):1260-73. doi: 10.1107/S0907444913015308. Epub 2013 Jun 18.
3. Evans P. Scaling and assessment of data quality. Acta Crystallogr D Biol Crystallogr. 2006 Jan;62(Pt 1):72-82.
4. De La Fortelle E, Bricogne G. Maximum-likelihood heavy-atom parameter refinement for the multiple isomorphous replacement and multiwavelength anomalous diffraction methods. Methods Enzym. 1997;276:473-94.
5. Vonrhein C, Blanc E, Roversi P, Bricogne G. Automated structure solution with autoSHARP. Methods Mol Biol. 2007;364:215-30.
6. Cowtan K. The Buccaneer software for automated model building. 1. Tracing protein chains. Acta Crystallogr D Biol Crystallogr. 2006 Sep;62(Pt 9):1002-11.
7. Emsley P, Cowtan K. Coot: model-building tools for molecular graphics. Acta Crystallogr D Biol Crystallogr. 2004 Dec;60(Pt 12 Pt 1):2126-32.
8. Murshudov GN, Vagin AA, Dodson EJ. Refinement of macromolecular structures by the maximum-likelihood method. Acta Crystallogr D Biol Crystallogr. 1997 May 1;53(Pt 3):240-55.
9. McCoy AJ, Grosse-Kunstleve RW, Adams PD, Winn MD, Storoni LC, Read RJ. Phaser crystallographic software. J Appl Crystallogr. 2007 Aug 1;40(Pt 4):658-674. Epub 2007 Jul 13.
10. Cutress ML, Whitaker HC, Mills IG, Stewart M, Neal DE. Structural basis for the nuclear import of the human androgen receptor. J Cell Sci. 2008 Apr 1;121(Pt 7):957-68. doi: 10.1242/jcs.022103. Epub 2008 Mar 4.
11. Sigrist CJ, Cerutti L, Hulo N, Gattiker A, Falquet L, Pagni M, Bairoch A, Bucher P. PROSITE: a documented database using patterns and profiles as motif descriptors. Brief Bioinform. 2002 Sep;3(3):265-74.

**Supplementary Figure legends**

Supplementary Figure 1. Conservation of CTNNBL1 in lower eukaryotes. A PSIPHRED secondary structural prediction of *S. pombe* CTNNBL1 homologue. Helical regions are represented as cylinders. The height of the blue rectangles above indicate confidence values. B Multiple sequence alignment of *S. pombe* CTNNBL1 to lower eukaryotic homologues.

Supplementary Figure 2. Multiple sequence alignment of CTNNBL1 across species.

Supplementary Figure 3. Purification of 6His-CTNNBL1Δ76. A. Comparison of purified 6His-CTNNBL1Δ76 at different concentrations (0.3, 1, 3 and 10 mg ml^-1^) using bovine serum albumin (BSA) as a reference shows the integrity of the protein used in pull down and ITC experiments. B. Circular dichroism analysis of purified His-CTNNBL1Δ76. The far-UV spectra of 6His-CTNNBL1Δ76 protein at 0.1 mg ml^-1^ in ITC buffer shows the predicted alpha-helical structure is maintained in solution at 20^0^ C. C Purified recombinant 6His-CTNNBL1Δ76 mutants analyzed by SDS/PAGE

Supplementary Figure 4. Logo representation of the karyopheryn-α armadillo repeats. The asterisk denotes the conserved WxxxN motif involved in NLS binding. Logo plots are derived from ProSite (11) as those presented in Figure 2B.

Supplementary Figure 5. Isothermal calorimetry plots superimposing 6His-CTNNBL1Δ76 (WT) (red) and 6His-CTNNBL1Δ76 mutants K7, K8 and K3 (grey) are shown with the characteristic binding to the CDC5L NLS3 peptide. All CTNNBL1 mutants displayed a complex binding pattern that was qualitatively similar, with Kd values equivalent to the wild type protein (WT), within error (K_d_1 values between ≥100 and 200 nM and K_d_2 between 2 and 12 μM). In the Δ521-563 deletion mutant (K_d_1 ~600 nM, K_d_2=30 μM) both Kd’s were reduced by a factor of 2-3. Similar results were obtained for the Prp31 peptide (with K_d_1 between ≥100 and 215 nM and K_d_2 between 1.6 and 27 μM).
