# Supplementary figures and images for: Structural and mutational analysis reveals that CTNNBL1 binds NLSs in a manner distinct from that of its closest armadillo-relative, karyopherin α
Source: FEBS Lett. 2014 Jan 3;588(1):21–7. doi: 10.1016/j.febslet.2013.11.013 (PMC3885797; doi:10.1016/j.febslet.2013.11.013)

*S. pombe* ctnnbl1 (SPAC1952.06c)

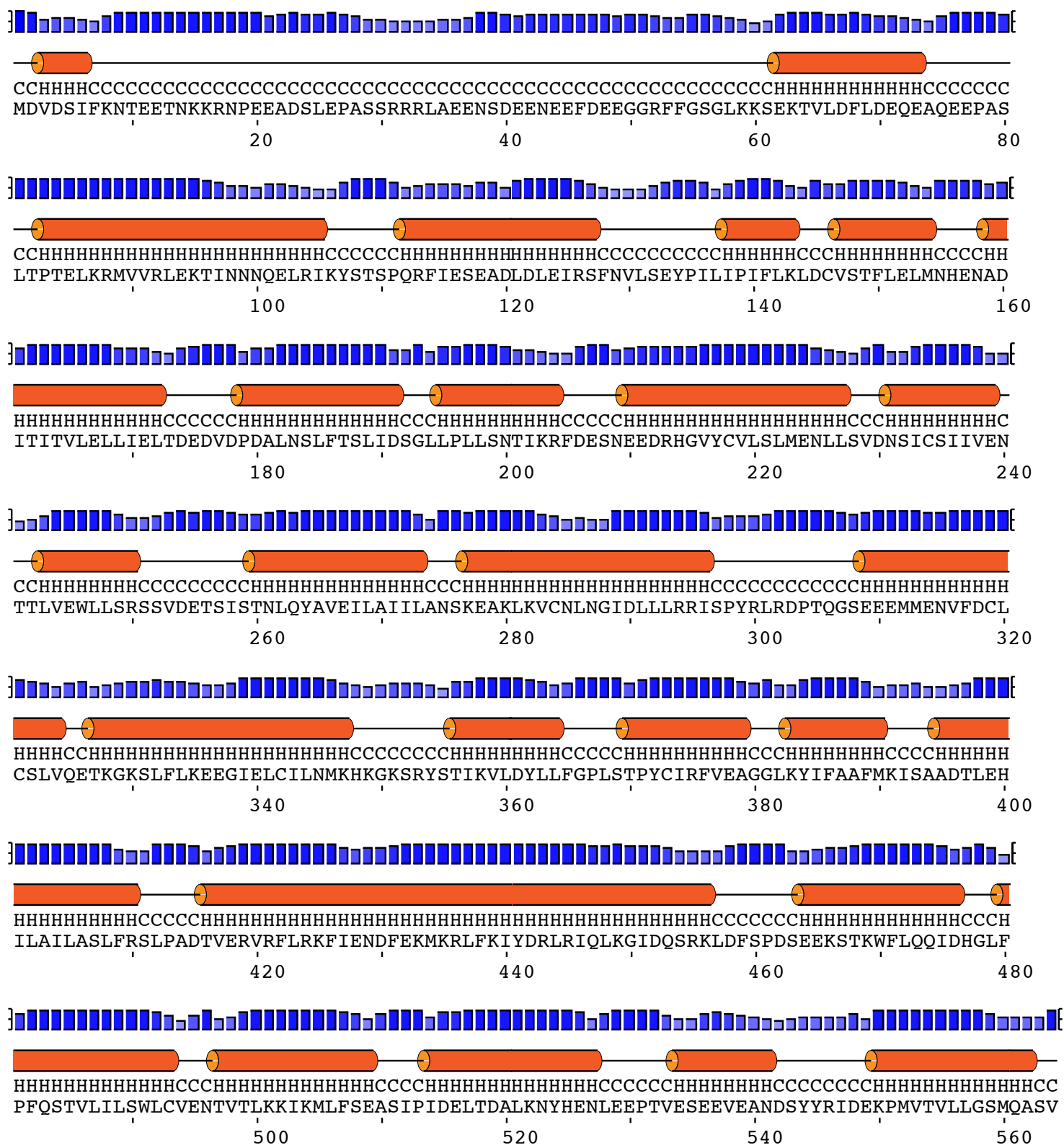

Supplement: Supplementary Fig. 1A [file mmc2.pdf]

**A**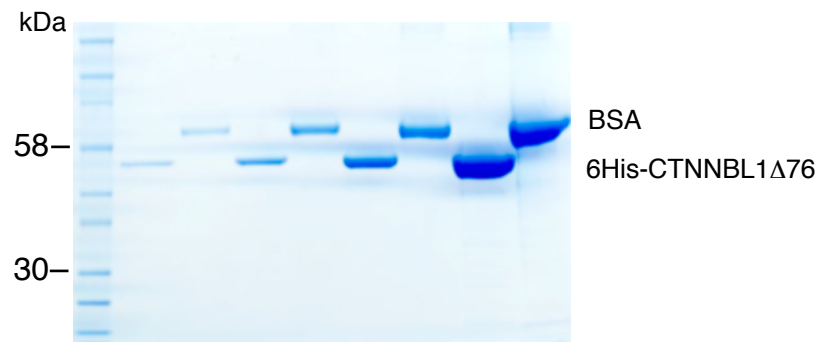**B**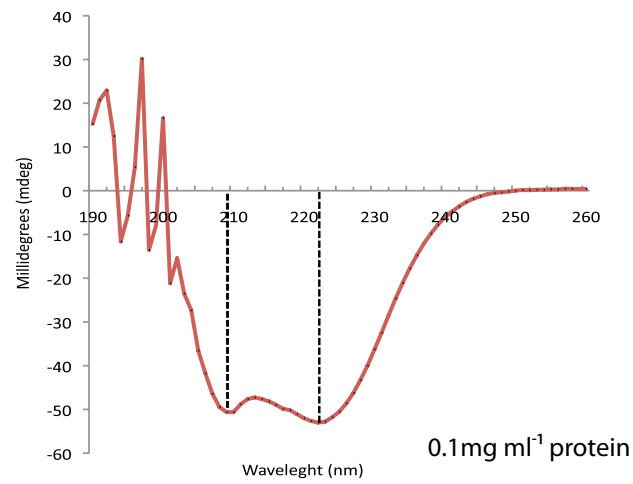**C**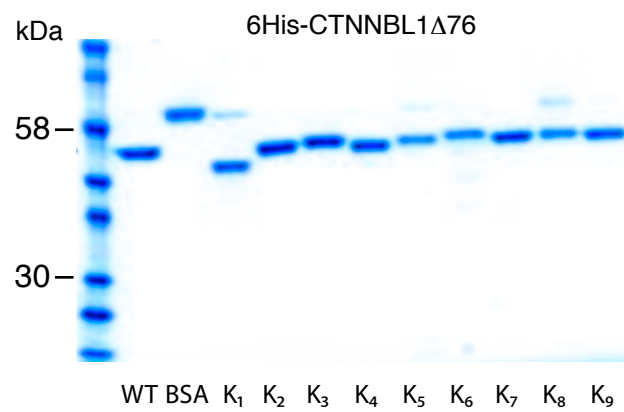

Supplement: Supplementary Fig. 3 [file mmc5.pdf]

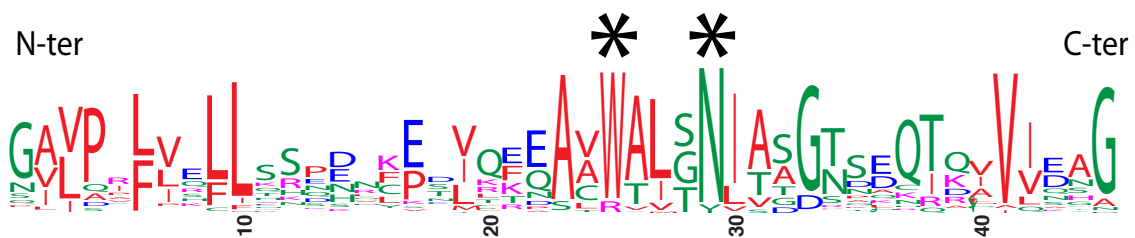

Supplement: Supplementary Fig. 4 [file mmc6.pdf]

# CDC5L NLS3

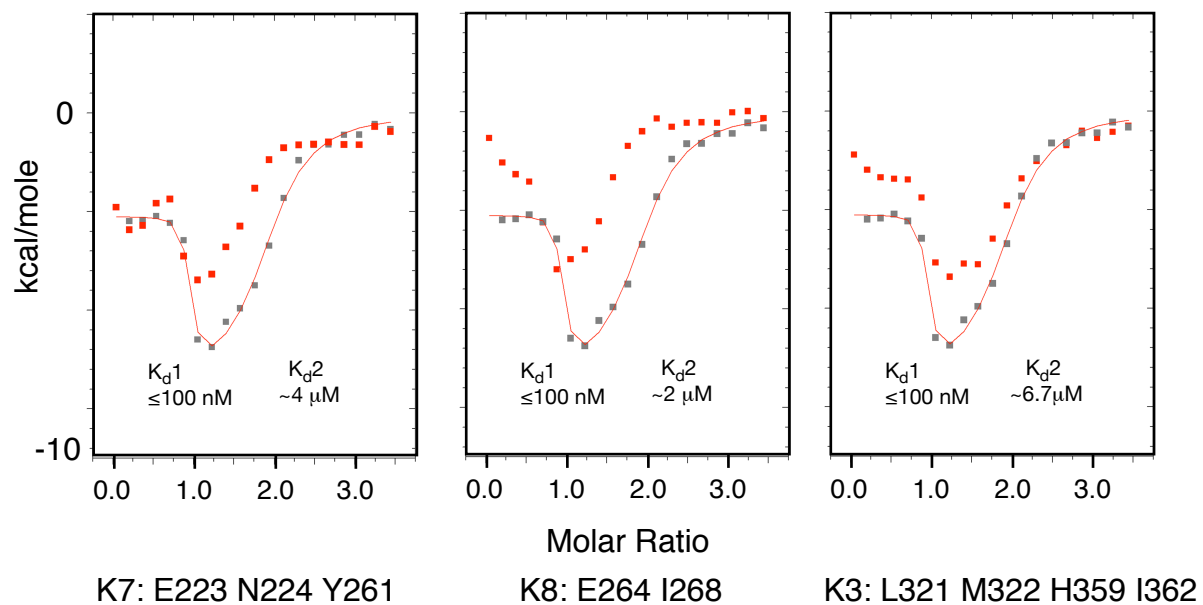

Supplement: Supplementary Fig. 5 [file mmc7.pdf]
