# Supplementary material for: Structural and mutational analysis reveals that CTNNBL1 binds NLSs in a manner distinct from that of its closest armadillo-relative, karyopherin α
Source: FEBS Lett. 2014 Jan 3;588(1):21–7. doi: 10.1016/j.febslet.2013.11.013 (PMC3885797; doi:10.1016/j.febslet.2013.11.013)
Supplement: Supplementary Fig. 1B [file mmc3.pdf]

Multiple sequence alignment of *S. pombe ctnnbl1* (SPAC1952.06c) within lower eukaryotes

```
S. pombe         1-43 M-DVDSIFKNTEETN------KKRNPEEAD---------SLEPAS----SRRRLAEENSDEEN-----------------
S. japonicus     1-43 M-NVDAIFKESNGPLd-----KKRKLEDGR----------------------YVGVDAENHDG--HPEQETks-------
C. posadasii     1-68 MtSIDELFKKPSLP-----sgQKRKFEPVRDPNEIYKSAKLDTNGaasgSRAATVDDAEDEDI-AGPELPP----DFG--
T. marneffei          --------------------------------------------------------------------------------
T. melanosporum  1-78 MaSLDNLFKKPALPSsssnssKKRKLESSVDPVAAYKSAKLEAPSndkgKSRAATVEEGDIDDtAGPSALPvnffDNA--
A. dermatitidis  1-67 MaSIDDLFKKPLLP-----s--KRKSEPLRDASALYKSAKVDANGdvssSRAAAVEDAPDEDNqVGPELPP----DFN--
N. crassa        1-66 M-NVDELFQKAGVPS-------KRKLDPIRDPNEIYKSAKLSSNG----SRHAQVEDGDDIE--AGPAPPPedgeDDGdy

S. pombe       44-110 --------EEFDEEGGRFFGSGLKKSEKTVLDFLDEQEAQEEPA-----SLTPTELKRMVVRLEKTINNNQELRIKYSTS
S. japonicus   44-113 -----KSeEEYDEEGGRFFGGGL--TEKEEKALNFLNTVDDGDN---EPTFDAAALKKKIIALEKALNKNQMLRTKYPNA
C. posadasii   69-138 -----QE-DIPDDEEGRFFGGGVTRDTT---NALDFIEQQEKEGVTA-GKIDSAWLKRTALNFEKRISKNAELRAKYESD
T. marneffei    1- 55 ----------------------MAKETA---QAMDYLDRQDQDGDTAVEKIDIAWVRRLALNFEKRVSKNAELRAKFEND
T. melanosporum 79-150 -----GE-EPEDEEGGRFFGGGVNKKTAEILDFMDERDRDDTAA--ASEKIDVAWLRRTALGFEKKINKNAEMRGKYEDD
A. dermatitidis 68-138 -----PDkDILDDEEGRFFGGGISRDTA---GALDFVEKLDQGDTRP-EKIDSAWLRRLALNFEKKISKNAELRAKFENE
N. crassa      67-146 gpsapPVeDDGDDEEGRFFGGGITAQEKEILDFMDNNTAAPADDLLTTDKIDLSWLKKTALNFEKRITRNAELRARHEDD

S. pombe       111-188 PQRFIESEADLDLEIRSFNVLSEYPILIPIFLKLDCVSTFLELMNHENADITITVLELLIELTDEDV--DPDALNSLFTS
S. japonicus   114-191 PEKFIESEADLDAEIHGLTVLSEYPELYSDLVQLNTTNTLFELFGHENIDIVIAIIDLLVELTDEDV--EPDSLIILQKD
C. posadasii   139-218 PQKFMASEADLDVDIKGLSILSEHPQLYPEFAELGCVGSLVSLLAHENTDIAIDVIEILAELTDEDVEAESEQWESLVDA
T. marneffei    56-135 PQKFMVSEADLDADIKSLSVLSEHPELYEEFAKLGCVASLVSLLAHENTDIAIDAIQTLTELTDEDVQAEQEHWDSLVNA
T. melanosporum 151-230 PHKFMASEADLDAGIKAISVLSEHPELFEEFRKLGCLASLVGLLAHENTDIAIDVVEVISELTDDEVEAEPEQWNAIVDG
A. dermatitidis 139-218 PQKFMESEADLDADIKSLSILSEHPDLYEEFASLGCVGSLVSLLSHENTDIAIDAIEILGELTDEDVEAEQEEWDVLVAA
N. crassa      147-226 PSKFIDSEADLDASIKALSILSEHPELYADFARLGCVSSLVSLLAHENTDIAIDAVEIINELTDEDVAASDEQFSALTDA

S. pombe       189-267 LIDSGLLPLLSNTIKRFDESNEEDRHGVYCVLSLMENLLSvDNSICSIIVENTTLVEWLLSRSSVDETS-ISTNLQYAVE
S. japonicus   192-269 LIEKGLFSIITEIMKRMNEENEDDAHGVFASFQLVENLVSiNADVCEEI-KNTDLIQFLLNRASKQEAT-VSENLQFSVE
C. posadasii   219-296 MLDADLIELLAQNLSRLDEDIESDRAGVYHILNVLENLSS-QSSVAEKIGQESEIMPWLYARIQKGEKS-VTQNKQYAAE
T. marneffei   136-213 MLDADVIELLAQNLSRLDESQDADRSGVYYVLNVLENLAS-QSSIAEKIGQDASIIPWLLSRIQQKETP-VGQNKQYSAE
T. melanosporum 231-308 MIEAQLLEMLTQNLSRLNEGNESDRNGVYHTLSVFENLAS-QQSLAEQMVRETNITPYLLQRIQARESP-ISQNKQYAAE
A. dermatitidis 219-296 MVDADVIALLSQNLARLDEGNDADRAGVYHVMSVLENFAS-QLPISEKIGQDPDMIPWLKGRIQKKETS-VSQNKQYAAE
N. crassa      227-305 LLEADLLGLLVSNFSRLDEQQEADRTGVYHALSIIENLCS-RRETADQIGKHTELLEWLLSRAKKSESPtVSQNKQYAAE

S. pombe       268-344 ILAIILANSKEAKLKVCN--LNGIDLLLRRISPYRLRDPTQGS-EEEMMENVFDCLCSLVQETKGKSLFLKEEGIELCIL
S. japonicus   270-346 LLAVLCSKSPAIRVSIVEk--NGIEILLNRISLYARRNPVPGL-EAELMQNAFDVLCSVVEEQQGKMQFLKEEGIELCLL
C. posadasii   297-374 VLAILLQSSQKNRERFAG--LNGVDTLLQLLSVYRKRDPEKDSDEEEYVENLFDSLTCVVDGALGKEKFVEAEGVELAQI
T. marneffei   214-291 ILAILLQSSSKNRNKFVS--LNGVDVLLQLLSSYRKRDPEKDSDEEEYVENLFDCLTCVVDEEDGKAKFLEAEGVELAQI
T. melanosporum 309-386 LLAILLQSSPANRKKLSE--LSGTDVLLQLLSPYRKRDPVKGGDEEEFVENVFDCLTCVVDELEGKEAFVDLEGVELVLI
A. dermatitidis 297-374 ILAILVQSSVKNRQRLLE--NNLVDVLLQLLSMYRKLDPEKDSDEEEYVENLFNCLTCLVDEDEGKAKFIDAEGVELAQI
N. crassa      306-385 ILAILVQSSPANRRRLASdeLNAVDTLLTLIAPYRRRDPERGSFEEEYMENLFECLTCLVDDPLGKTKFVEAEGVELCLL

S. pombe       345-402 NMKh---KGK-SRYSTIKVLDYLLFGPLSTPYCIRFVEAGGLKYIFAAFM----------KISAa--------DTLEHIL
S. japonicus   347-404 MLQqk----KQSRKPAFKLVDHALFGPLSLPLCNRFVEFGGLKYLFSTFM---------KKMEA---------EMLEHIC
C. posadasii   375-432 MLR----EGKMSKPRALRVLNHAVGGKDGARVCEQLVEAGLLRTVFGMFM---------KKQDN---------QTVEHLL
T. marneffei   292-349 MLR----EGKLSKQRALRTLDHALSGQTGAAACDRLIEVAGLRTVFGMFM---------KKQEK---------EAMEHLL
T. melanosporum 387-444 MLR----EGKMSKPRALRLLDHAVGGQSGANVCQKLVDAAGLKTVFGMFM---------KKQDN---------QTTEHLL
A. dermatitidis 375-432 MLR----EGKMSKPRALRVLSHAVGGKGGASVCERLIEAALLGTIFGMFM---------KNQDH---------QSTEHLL
N. crassa      386-463 ILSsadiKGKLSKPACLRLLDHAAS--FSSEVCLKIVEAGGLKTLFTLFMsgdkssdstKKKNKggktptlskQDTEHLL

S. pombe       403-461 AILASLFRSLPADTVE----------------RVRFLRKFIENDFEKMKRLFKIYDRLRIQLKGIDQS-----RKLDFSp
S. japonicus   405-466 AIMASLFRSLAADTPE----------------RIRFLAKFVEKDFTKTKKLVDFYSKLRKPITEIRQSAKAQ-TNLEED-
C. posadasii   433-495 GIFASLLRLLPGESAG----------------RIRTLAKFVEKDYEKVSRLVQFRRGYASKLLPIDQAIAQERANLSKD-
T. marneffei   350-412 GIFASLLRHLPGGSAP----------------RIRTLAKFMEKDYEKIEKLIKLRREYSSRLSPVESGIEQERQGLDES-
T. melanosporum 445-508 GIFAALLTHLPADSAS----------------RIRTLAKFVEKDYEKIAKLTKLRTDYALRVAKVDAEIAVQREDLGSVv
A. dermatitidis 433-495 GIFSSLLRLLPGESSS----------------RIRTLAKFMEKDYEKIKKLVKLRWEYASKVSEVDQLIHQERKSMSSE-
N. crassa      464-543 GIFASMLRHLPATSSSppsssvtddsetsgaeRIRLLAKFVEKNYAKTSKLIRLRREYAARVSQVDEAIKADAASSPEQf

S. pombe       462-541 DSEEKSTKWFLQQIDHGLFPFQSTVLILSWLCVENTVTLKKIKMLFSEASIPIDELTDALKNYHENLEEPTVEseevean
S. japonicus   467-541 -----SLALFLKQIDMGLFSFQSIVVILAWLCCEDSAICECISNSLKAFGLQLHDLLSDLQDYFENFAESPETisdekse
C. posadasii   496-567 EQDAMAVEWLSRRLDVGLFSLQIIDVILAWLVAEDDGAKARIKSLLSDQDQDLSIIRATLEEQLSGL-EGPEG-------
T. marneffei   413-485 DQEIMAGEWLSRRLDAGLFALQTIDVILAWLIAEDDGAKTKISALLGDRDEDISLIGKTLQDQVNDLGDEDEG-------
T. melanosporum 509-581 EEEEMAEEWFSKRLDAGLFCLQMTDRILAWLCAEDDGAKKRIGGLLGRAGGSLRDVRRTLKEQIDGMTEASDE-------
A. dermatitidis 496-567 EQELMSLEWLSRRLDAGLFCLQTIDVILAWLVAEDDGAKAKVKSLLSDRDEDLAIVKTTLQEQLNGL-DETST-------
N. crassa      544-612 DED----EAFSRRLDAGLFCLQTIDVILAWLIAEDTGCRAKIRELLKDRDEDFGVLARTLREQMAGVDSETEE-------

S. pombe       542-564 dsyYRIDEKPMVTVLLGSMQasv------------
S. japonicus   542-576 esiPRVDEKPMVQTLLEIMQiqvhesqetnsgrsa
C. posadasii   568-582 ---EE-EEKDMLTTLLEFI----------------
T. marneffei   486-501 ---EK-DLKDMLSTLLQFVQ---------------
T. melanosporum 582-597 ---WQQVIKDMLSTLIEFL----------------
A. dermatitidis 568-583 ---DELDTREMLGTLLLFL----------------
N. crassa      613-628 ---GR-DTGEMLKALLEFLE---------------
```
