# Supplementary material for: Structural and mutational analysis reveals that CTNNBL1 binds NLSs in a manner distinct from that of its closest armadillo-relative, karyopherin α
Source: FEBS Lett. 2014 Jan 3;588(1):21–7. doi: 10.1016/j.febslet.2013.11.013 (PMC3885797; doi:10.1016/j.febslet.2013.11.013)
Supplement: Supplementary Fig. 2 [file mmc4.pdf]

Supplementary Figure 2. Multiple sequence alignment of CTNNBL1 homologues based on UniProt/SwissProt family asigment

```
Anole Lizard (Anolis carolinensis)   MDVGELLSYQPNRGTKRSREDEE------EDLK--V-RHKHSG--------------------------------------GAR--------------ERGR--HREEEPP---------
Cow (Bos taurus)                     MDVGELLSYQPNRGTKRPRDDEE------EELK--M-RRRQA---------------------------------------GTR--------------ERGR--YREEEMT---------
Marmoset (Callithrix jacchus)        MDVGELLSYQPNRGTKRPRDDEE------EEQK--M-RRKQT---------------------------------------GTR--------------ERSR--YREEEMT---------
Dog (Canis familiaris)               MDVGELLSYQPNRGTKRPRDDEE------EELK--M-RRRQA---------------------------------------GPR--------------DRGR--YREEEMT---------
Guinea Pig (Cavia porcellus)         MDVGELLSYQPNRGTKRPRDEEE------EEQK--T-RRKQA---------------------------------------GPR--------------ERGR--YREEEVT---------
Seasquirt (Ciona intestinalis)       MDVGELLAFKPKTGKR-DYDDAPT-----ESPKPKIS---KPALK------------------------PKKKATRLQLQAGAAVSSGLKRSGGSAPASSAV--SPLEE-----------
Pacific Seasquirt (Ciona savignyi)   MDVGELLAFKPQTGKR-EYDDGAT-----EQPKTKVV---KSALKSDKSMEKELDTFFVNAKLGVTGERPKRKATRLQLQSGAPI-------------SSGV--KRSEN-----------
Zebrafish (Danio rerio)              MDVGELLNYQPDRGAKRPRDGDS------HEES-RS-KQK-A---------------------------------------AGR--------------DAGRVLLRSEDNT---------
Fruitfly (Drosophila melanogaster)   MDIGELLAFKPEQTPKRPHDDED------DEFDLASSHGKRGGVD------------------------E--KTKRMRRIADAK--------------ESAH--YVKQSGGATGGSGTSA
Horse (Equus caballus)               MDVGELLSYQPNRGTKRPRDDEE------EELK--M-RRKQA---------------------------------------GTR--------------ERGR--YREEEMTA--------
Chicken (Gallus gallus)              MDVGELLSYQPNRGTKRPRDEEE------EEIK--A-RRKQV---------------------------------------SSR--------------EHGR--HREEESA---------
Stickleback (Gasterosteus aculeatus) MDVGELLNYQPDRGAKRPRGEDGGAAGGGEETRGGK-QQKGL---------------------------------------SAR--------------ELAR--YREAATAGM------E
Gorilla (Gorilla gorilla)            MDVGELLSYQPNRGTKRPRDDEE------EEQK--M-RRKQT---------------------------------------GTR--------------ERGR--YREEEMT---------
Human (Homo sapiens)                 MDVGELLSYQPNRGTKRPRDDEE------EEQK--M-RRKQT---------------------------------------GTR--------------ERGR--YREEEMT---------
Elephant (Loxodonta africana)        MDVGELLSYQPNRGTKRPRDDEE------EELK--M-RRKQA---------------------------------------GTR------------ERERGR--HREEEMT---------
Opossum (Monodelphis domestica)      MDVGELLNYQPNRGTKRPRDDEE------EELK--T-RRKQA---------------------------------------GAR--------------ERSR--YREEDGA---------
Mouse (Mus musculus)                 MDVGELLSYQPNRGTKRPRDDEE------EELK--T-RRKQT---------------------------------------GPR--------------ERGR--YREEEAT---------
Microbat (Myotis lucifugus)          MDVGELLSYQPNSGTKRPRDDEE------EELQ--M-RRKQA---------------------------------------GTR--------------ERGR--FREEEMT---------
Pika (Ochotona princeps)             MDVGELLSYQPNRGTKRPRDDEE------EELK--T-RRKQA---------------------------------------GPR--------------ERGR--FREEELT---------
Rabbit (Oryctolagus cuniculus)       MDVGELLSYQPNRGTKRPRDDEE------EELK--T-RRKQA---------------------------------------GPR--------------ERGR--YREEELT---------
Medaka (Oryzias latipes)             MDVGELLNYQPDRGAKRPREQEGRAAGGDDDSRGGK-QQKGL---------------------------------------GSR--------------DPAR--HREAGAGSEG-R---A
Chimpanzee (Pan troglodytes)         MDVGELLSYQPNRGTKRPRDDEE------EEQK--M-RRKQT---------------------------------------GTR--------------ERGR--YREEEMT---------
Orangutan (Pongo pygmaeus)           MDVGELLSYQPNRGTKRPRDDEE------EEQK--M-RRKQT---------------------------------------GTR--------------ERGR--YREEEMT---------
Rat (Rattus norvegicus)              MDLSSFLFSQPNRGTKRPRDDEE------EELK--T-RRKQT---------------------------------------GPR--------------ERGR--YREDEAT---------
Zebra Finch (Taeniopygia guttata)    MDVGELLSYQPNRSTKRPRDEEE------EEIK--A-RRKQA---------------------------------------SAR--------------EHGR--HREEESA---------
Fugu (Takifugu rubripes)             MDVGELLKYQPDRGAKRPRAEEKGAALGDEESR--G-GKHQK---------------------------------------G----------------HSAR--YREAASLAIEIG---G
Tetraodon (Tetraodon nigroviridis)   MDVGELLKYQPDRGAKRPRGEEKGGSAGDDESR--G-VKHQK---------------------------------------G----------------HSAR--YREAA-----------
Dolphin (Tursiops truncatus)         MDVGELLSYQPNRGTKRPRDDEE------EELK--M-RRRQA---------------------------------------GTR--------------ERGR--FREEEMT---------
Xenopus tropicalis                   MDVGELLRYQPDRGTKRGRDDEP------EDLR--P-PRKER---------------------------------------GGR--------------NWHR----DEERGP--------
                                     **:..:*  :*.   :             ..
```

```
Anole Lizard (Anolis carolinensis)    ---AAEETEDEKKKLL-QIIEK-GD-EEE-EEDEPLDESSVKKMILTFEKRSYKNQELRIKFPDNPEK--FMESELDLNDIIQEMHVIATMPDLYHLLVELNAVHSLLGLLGHDNTD-I-
Cow (Bos taurus)                      ---VVEEADDDKKRLL-QIIDREGE-EEE-EEEEPLDESSVKKMILTFEKRSYKNQELRIKFPDNPEK--FMESELDLNDIIQEMHVVATMPDLYHLLVELNAVQSLLGLLGHDNTD-V-
Marmoset (Callithrix jacchus)         ---VVEEADDDKKRLL-QIIDRDGE-EEE-EEEEPLDESSVKKMILTFEKRSYKNQELRIKFPDNPEK--FMESELDLNDIIQEMHVVATMPDLYHLLVELNAVQSLLGLLGHDNTD-V-
Dog (Canis familiaris)                ---VVEEADDDKKRLL-QIIDREGE-EEE-EEEEPLDESSVKKMILTFEKRSYKNQELRIKFPDNPEK--FMESELDLNDIIQEMHVVATMPDLYHLLVELNAVQSLLGLLGHDNTD-I-
Guinea Pig (Cavia porcellus)          ---VAEEADDDKKRLL-QIIDREGE-EEE-EEEEPLDESSVKKMILTFEKRSYKNQELRIKFPDNPEK--FMESELDLNDIIQEMHVVATMPDLYHLLVELNAVQSLLGLLGHDNTD-V-
Seasquirt (Ciona intestinalis)        -NVNDETEEERQMKLL-EMWEEKVKAKDL-NDEEQYDDQKVKKLILSFEKKVYKNQEMRIKFPDNPEK--FMESELDLNDVVMEMKILATMPEQYHYLVELRSVNSLLALINHANTD-I-
Pacific Seasquirt (Ciona savignyi)    -EI-DETEEERQMKLL-EMWEEKVKAKEI-NEEEQYDDQKVKKLILTFEKKVYKNQEMRIKFPDNPEK--FMESELDLNDVIVEMKILATLPEQYHYLVELRAVNSLLTLINHANTD-I-
Zebrafish (Danio rerio)               ---EEPTAGDGDKVLE-RLMDQDGE-D---PEAELVDESTVKKMILTFEKRSYKNQELRIKFPDNPEK--FMEAELDLNDIIQEMHVIATIPELYHLLVELNAVQSLLGLLSHDNTD-I-
Fruitfly (Drosophila melanogaster)    FEFDPELTEEERLNIL-----KYVE-NEE-ADGDVLDEQSLKKLILVFEKRNLKNQEMRIKFADSPEK--FMDSEVELHTVIQELKGVATVPDLYPILVDLHGLHSLLELLAHQNTD-I-
Horse (Equus caballus)                ---VVEEADDDKKRLL-QIIDREGE-EEE-EEEEPLDESSVKKMILTFEKRSYKNQELRIKFPDNPEK--FMESELDLNDIIQEMHVVATMPDLYHLLVELNAVQSLLGLLGHDNTD-I-
Chicken (Gallus gallus)               ---VQEETDDEKKKLL-QIIERDGE-EED-EEEEPLDESSVKKMILTFEKRSYKNQELRIKFPDNPEK--FMESELDLNDIIQEMHVIATMPDLYHLLVELNAVQSLLGLLGHDNTAHV-
Stickleback (Gasterosteus aculeatus) GGGGAEDLTGDKKRILEKLMDRD---EEE-PEAEPVDESSVKKMILTFEKRSYKNQELRIKFPDNPEK--FMESELDLNDIIQEMHVIATMPDLYHLLVELNAVHSLLGLLSHENTD-V-
Gorilla (Gorilla gorilla)             ---VVEEADDDKKRLL-QIIDRDGE-EEE-EEEEPLDESSVKKMILTFEKRSYKNQELRIKFPDNPEK--FMESELDLNDIIQEMHVVATMPDLYHLLVELNAVQSLLGLLGHDNTD-V-
Human (Homo sapiens)                  ---VVEEADDDKKRLL-QIIDRDGE-EEE-EEEEPLDESSVKKMILTFEKRSYKNQELRIKFPDNPEK--FMESELDLNDIIQEMHVVATMPDLYHLLVELNAVQSLLGLLGHDNTD-V-
Elephant (Loxodonta africana)         ---VVEEADDDKKRLL-QIIDREGE-EEE-EEEEPLDESSVKKMILTFEKRSYKNQELRIKFPDNPEK--FMESELDLNDIIQEMHVVATMPDLYHLLVELNAVQSLLGLLGHDNTD-V-
Opossum (Monodelphis domestica)       ---AVEEADDDKKKLL-QMIDKEGE-EEE-EEDEPLDESSVKKMILTFEKRSYKNQELRIKFPDNPEK--FMESELDLNDIIQEMHVVATMPDLYHLLVELNAVQSLLGLLGHDNTD-IL
Mouse (Mus musculus)                  ---AAEDTADDKKRLL-QIIDRDGE-EEE-EEEEPLDESSVKKMILTFEKRSYKNQELRIKFPDNPEK--FMESELDLNDIIQEMHVVATMPDLYHLLVELSAVQSLLGLLGHDNTD-V-
Microbat (Myotis lucifugus)           ---VVEEADDDKKRLL-QIIDREGE-EEE-EEEEPLDESSVKKMILTFEKRSYKNQELRIKFPDNPEK--FMESELDLNDIIQEMHVVATMPDLYHLLVELNAVQSLLGLLGHDNTD-I-
Pika (Ochotona princeps)              ---VAEEADDDKKRLL-QIIDRDGE-EEE-EEEEPLDESSVKKMILTFEKRSYKNQELRIKFPDNPEK--FMESELDLNDIIQEMHVVATMPDLYHLLVELNAVQSLLGLLGHDNTD-V-
Rabbit (Oryctolagus cuniculus)        ---VVEEADDDKKRLL-QIIDRDGE-DEE-EEEEPLDESSVKKMILTFEKRSYKNQELRIKFPDNPEK--FMESELDLNDIIQEMHVVATMPDLYHLLVELNAVQSLLGLLGHDNTD-V-
Medaka (Oryzias latipes)              EPGDGEEQTGDKKRILEKLMDQDEE-EPEIKQAEPVDESTVKKMILTFEKRSYKNQELRIKFPDNPEKTRFMESELDLNDIIQEMHVIATMPDLYHLLVELNAVHSLLGLLSHENTAHV-
Chimpanzee (Pan troglodytes)          ---VVEEADDDKKRLL-QIIDRDGE-EEE-EEEEPLDESSVKKMILTFEKRSYKNQELRIKFPDNPEK--FMESELDLNDIIQEMHVVATMPDLYHLLVELNAVQSLLGLLGHDNTD-V-
Orangutan (Pongo pygmaeus)            ---VVEEADDDKKRLL-QIIDRDGE-EEE-EEEEPLDESSVKKMILTFEKRSYKNQELRIKFPDNPEK--FMESELDLNDIIQEMHVVATMPDLYHLLVELNAVQSLLGLLGHDNTD-V-
Rat (Rattus norvegicus)               ---AAEDADDDKKRLL-QIIDRDGE-EEE-EEEEPLDESSVKKMILTFEKRSYKNQELRIKFPDNPEK--FMESELDLNDIIQEMHVVATMPDLYHLLVELSAVQSLLGLLGHDNTD-V-
Zebra Finch (Taeniopygia guttata)     ---ALEETDDEKKKLL-QIIERDGE-EED-EEEEPLDESSVKKMILTFEKRSYKNQELRIKFPDNPEK--FMESELDLNDIIQEMHVIATMPGLYHLLVELNAVQSLLGLLGHDNTD-I-
Fugu (Takifugu rubripes)              EMRENEEMNEEKKIILEKLLDRD---EEE-PEAEPVDESSVKKMILTFEKRSYKNQELRIKFPDSPEK--FMESELDLNDIIQEMHVIATIPELYHLLVELHAVQSLLGLLSHENTD-I-
Tetraodon (Tetraodon nigroviridis)    -SMGIEAGG--------KLLDRDDE-NRV-KQAEPVDESSVKKMILTFEKRSYKNQELRIKFPDSPEK--FMESELDLNDIIQEMHVIATIPDLYHLLVELHAVQSLLGLLSHENTD-IL
Dolphin (Tursiops truncatus)          ---VVEEADDDKKRLL-QIIDREGE-EEE-EEEEPLDESSVKKMILTFEKRSYKNQELRIKFPDNPEK--FMESELDLNDIIQEMHVVATMPDLYHLLVELNAVQSLLGLLGHDNTD-V-
Xenopus tropicalis                    --TAEE----DRNA-----LD-DGD--DE-EENETLDEGAVKRMILNFEKRSYKNQELRIKFPDNPEK--FMESELDLNDIIQEMHVIATIPDLYHLLVELNAVQSLLGFLPH-----V-
                                                                     : :  *:  :*::** ***:  ****:****.*.***  **::*::*: :: *:: :**:*  *  **:* .::*** :: *     :
```

```
Anole Lizard (Anolis carolinensis)   --------LS---VMALRRQLTDIDTLHESEEGAEVLIDAL--VDGQVVALLVQNLERLDENVKEEADGVHNTLAIIENMAEFRPEMCSDAAQQGLMQWLLKRLKAKMPFDANKLYCSEV
Cow (Bos taurus)                     ----S---IA---VVDLLQELTDIDTLHESEEGAEVLTDAL--VDGQVVALLVQNLERLDESVKEEADGVHNTLAIVENMAEFRPEMCTEAAQQGLLQWLLKRLKAKMPFDANKLYCSEV
Marmoset (Callithrix jacchus)        ----S---IA---VVDLLQELTDIDTLHESEEGAEVLIDAL--VDGQVVALLVQNLERLDESVKEEADGVHNTLAIVENMAEFRPEMCTEGAQQGLLQWLLKRLKAKMPFDANKLYCSEV
Dog (Canis familiaris)               ----L---SA---VVDLLQELTDIDTLHESEEGAEVLIDALLKVDGQVVALLVQNLERLDESVKEEADGVHNTLAIVENMAEFRPEMCTEAAQQGLLQWLLKRLKAKMPFDANKLYCSEV
Guinea Pig (Cavia porcellus)         ----S---IA---VVDLLQELTDIDTLHESEEGAEVLIDAL--VDGQVVALLVQNLERLDESVREEADGVHNTLAIVENMAEFRPEMCTEAAQQGLLQWLLKRLKAKMPFDANKLYCSEV
Seasquirt (Ciona intestinalis)       ----A---IA---VIDLIQELTEVDSVTENEEGANLLADAL--MDGQAGSLIVQNLERLDESVKEEATGVHNSLGVVENLLELKPEFGLNFAQQGFVAWLLKRLKAKIPFSGNKLYASEI
Pacific Seasquirt (Ciona savignyi)   ----A---IA---VVDLMQELTEVDSNSENEEGAILLADAL--VEGQVGSLLVQNLERLDESVKEEASGVHNSLGVIENMLELKPEFGMSFAQQGLVTWLLKRLKAKMPFDANKLYTCET
Zebrafish (Danio rerio)              ----M---TSQSQLFAMITELTDIDTLHESEEGAEVLIDAL--LEGQVVALMVQNMERLDETVKEEADGVYNTLAVIENMAEFRPGLCTEAAQQGLMQWLLKRIKAKMPFDANKLYCSEI
Fruitfly (Drosophila melanogaster)   ----A---VA---VVDLLQEVTDVDILGESQEGAESLIEAL--RKEQICALLVQNLERLNEQVKEEADGVHNTLAIVENLTEIDSEFVKEAAEQGLLAWLLKRLRGKSPFDPNKLYCSEI
Horse (Equus caballus)               ----L---SA---VVDLLQELTDIDTLHESEEGAEVLIDAL--VDGQVVALLVQNLERLDESVKEEADGVHNTLAIVENMAEFRPEMCTEAAQQGLLQWLLKRLKAKMPFDANKLYCSEV
Chicken (Gallus gallus)              ----S---IA---VVDLLQELTDIDTLHESEEGAEVLIDAL--VEGQVVALLVQNLERLDESVKEEADGVHNTLAIVENMAEFRPEMCTEAAQQGLMQWLLKRLKAKMPFDANKLYCSEV
Stickleback (Gasterosteus aculeatus)  ----A---IA---VVDLLQELTDIDTLQESEEGAEVLIDSL--LEGQVVALLVQNMERLDEQVKEEADGIYNTLAITENMAEFRPGLCTEAAQQGLMQWLLKRIKAKIPFDANKLYCSEI
Gorilla (Gorilla gorilla)            ----S---IA---VVDLLQELTDIDTLHESEEGAEVLIDAL--VDGQVVALLVQNLERLDESVKEEADGVHNTLAIVENMAEFRPEMCTEGAQQGLLQWLLKRLKAKMPFDANKLYCSEV
Human (Homo sapiens)                 ----S---IA---VVDLLQELTDIDTLHESEEGAEVLIDAL--VDGQVVALLVQNLERLDESVKEEADGVHNTLAIVENMAEFRPEMCTEGAQQGLLQWLLKRLKAKMPFDANKLYCSEV
Elephant (Loxodonta africana)        ----S---IA---VVDLLQELTDIDTLHESEEGAEVLIDAL--VDGQVVALLVQNLERLDESVKEEADGVHNTLAIVENMAEFRPEVCAEAAQQGLLQWLLKRLKAKMPFDANKLYCSEV
Opossum (Monodelphis domestica)      TFLVS---IA---VVDLLQELTDIDTLHESEEGAEVLIDAL--VDGQVAALLVQNLERLDESVKEEADGVHNTLAIVENMAEFRPEMCTEAAQQGLMQWLLKRLKAKMPFDANKLYCSEV
Mouse (Mus musculus)                 ----S---IA---VVDLLQELTDIDTLHESEEGAEVLIDAL--VDGQVAALLVQNLERLDESVREEADGVHNTLAIVENMAEFRPEMCTEAAQQGLLQWLLKRLKAKMPFDANKLYCSEV
Microbat (Myotis lucifugus)          ----LSDLSS---VVDLLQELTDIDTLHESEEGAEVLIDAL--VDGQVVALLVQNLERLDESVKEEADGVHNTLAIVENMAEFRPEMCTEAAQQGLLQWLLKRLKAKMPFDANKLYCSEV
Pika (Ochotona princeps)             ----S---IA---VVDLLQELTDIDTLHESEEGAEVLIDAL--VDGQVVALLVQNLERLDESVREEADGVHNTLAIVENMAEFRPEMCTEAAQQGLLQWLLKRLKAKMPFDANKLYCSEV
Rabbit (Oryctolagus cuniculus)       ----S---IA---VVDLLQELTDIDTLHESEEGAEVLIDAL--VDGQVVALLVQNLERLDESVREEADGVHNTLAIVENMAEFRPEMCTEAAQQGLLQWLLKRLKAKMPFDANKLYCSEV
Medaka (Oryzias latipes)             ----A---IA---VVDLLQELTDIDTLHESEEGAEVLIDAL--LEGQVVALLVQNMERLDEQVKEESDGIYNTLAIIENMAEFRPGLCAEAAQQGLMQWLLKRIKAKMPFDANKLYCSEI
Chimpanzee (Pan troglodytes)         ----S---IA---VVDLLQELTDIDTLHESEEGAEVLIDAL--VDGQVVALLVQNLERLDESVKEEADGVHNTLAIVENMAEFRPEMCTEGAQQGLLQWLLKRLKAKMPFDANKLYCSEV
Orangutan (Pongo pygmaeus)           ----S---IA---VVDLLQELTDIDTLHESEEGAEVLIDAL--VDGQVVALLVQNLERLDESVKEEADGVHNTLAIVENMAEFRPEMCTEGAQQGLLQWLLKRLKAKMPFDANKLYCSEV
Rat (Rattus norvegicus)              ----S---IA---VVDLLQELTDIDTLHESEEGAEVLIDAL--VDGQVVALLVQNLERLDESVREEADGVHNTLAIVENMAEFRPEMCTEAAQQGLLQWLLTRLKAKMPFDANKLYCSEV
Zebra Finch (Taeniopygia guttata)    ----L---HT---VVDLLQELTDIDTLHESEEGAEVLIDAL--VEGQVVALLVQNLERLDESVKEEADGVHNTLGIVENMAEFRPEMCTEAAQQGLMQWLLKRLKAKMPFDANKLYCSEV
Fugu (Takifugu rubripes)             ----L---SS---VVDLLQELTDIDTLHESEEGAEVLIDAL--LEGQVVALLVQNMERLDETVKEEGDGVHNTLAIIENMAEFRPGLCTEAAQQGLMQWLLKRIKAKMPFDANKLYCSEI
Tetraodon (Tetraodon nigroviridis)   K-HVA---IA---VVDLLQELTDIDTLHESEEGAEVLIDAL--LEGQVVALLVQNMERLDETVKEEADGVHNTLAIVENMAEFRPGLCTEAAQQGLMQWLLKRIKAKMPFDANKLYSSEI
Dolphin (Tursiops truncatus)         ----S---IA---VVDLLQELTDIDTLHESEEGAEVLIDAL--VDGQVVALLVQNLERLDESVKEEADGVHNTLAIVENMAEFRPEMCTEAAQQGLLQWLLKRLKAKMPFDANKLYCSEV
Xenopus tropicalis                   ----C---IA---VVDLLQELTDIDTLHESEEGAQVLIDAL--LEGQVVALLVQNLERLDESTKEEADGVHNTLAIIENMTEFRPEICSDAAQQGLLHWLLKRLKAKMPFDANKLYCSEI
                                              :   :. :  ::*::*   *.:***  * ::*   . *  :*:***:***:* .:**. *::*:*.: **: *: . .  . *:**:: ***.*::.* **. **** .*
```

```
Anole Lizard (Anolis carolinensis)   LAILLQNKDDNRELLGELDGIDVLLQQLSVFKRHNPSTAEEQEMMENLFDSLCSSLMLSSNRERFLKGEGLQLMNLMLREKKISRSSALKVLDHAMIGPEGADNCHKFVDILGLRTIFPL
Cow (Bos taurus)                     LAILLQDNDENRELLGELDGIDVLLQQLSVFKRHNPSTAEEQEMMENLFDSLCSCLMLSSNRERFLKGEGLQLMNLMLREKKISRSSALKVLDHAMIGPEGTDNCHKFVDILGLRTIFPL
Marmoset (Callithrix jacchus)        LAILLQDNDENRELLGELDGIDVLLQQLSVFKRHNPSTAEEQEMMENLFDSLCSCLMLSSNRERFLKGEGLQLMNLMLREKKISRSSALKVLDHAMIGPEGTDNCHKFVDILGLRTIFPL
Dog (Canis familiaris)               LAILLQDNDENRELLGELDGIDVLLQQLSVFKRHNPSTAEEQEMMENLFDSLCSCLMLSSNRERFLKGEGLQLMNLMLREKKISRSSALKVLDHAMIGPEGTDNCHKFVDILGLRTIFPL
Guinea Pig (Cavia porcellus)         LAILLQDNDENRELLGELDGIDVLLQQLSVFKRHNPSTAEEQEMMENLFDSLCSCLMLSSNRERFLKGEGLQLMNLMLREKKISRSSALKVLDHAMIGPEGTDNCHKFVDILGLRTIFPL
Seasquirt (Ciona intestinalis)       LSILLQSHDEIRKLVGELEGIDILLQQLAAFKRHDPKGAEEVEFMENLFNCLCSSLLLVDNRSKFLHGEGLQLMNLMLREKKMSRNSALKVLDHAMSGPDGASNSAKFVDILGLRTLFPI
Pacific Seasquirt (Ciona savignyi)   LSILLQQNDEIRKLLGESDGIDILLQQLAAFKRHDPKGAEEVEFMENLFNCLCSALLLASNRGKFLQGEGLQLMNLMLREKKMSRNSALKVLDHAMSGPDGASNSAKFVDILGLRTLFPI
Zebrafish (Danio rerio)              LAILLQNNDKTRELLGEMDGIDVLLQQLSVFKRHNPSTAEEQEMMENLFDALCSCLMLPANRDRFLRGEGLQLMNLMLREKKMSRVSALKVLDYGMIGPEGSDNCHKFVDILGLRTIFPL
Fruitfly (Drosophila melanogaster)   LSILIQTENENRLLLGSLDGVDVLLQQLAVYKRHDPASNEEQEYMQNLFNCLCSALMARENRDRFLSGEGLQLMNLMLREKKMSRNGSLKVLDHAMAGQDGRDNCNKFVEILGLRTIFPL
Horse (Equus caballus)               LAILLQDNDENRELLGELDGIDVLLQQLSVFKRHNPSTAEEQEMMENLFDSLCSCLMLSSNRERFLKGEGLQLMNLMLREKKISRSSALKVLDHAMIGPEGTDNCHKFVDILGLRTIFPL
Chicken (Gallus gallus)              LAILLQNNDDNRELLGELDGIDVLLQQLSVFKRHNPSTAEEQEMMENLFDSLCSCLMLSSNRDRFLKGEGLQLMNLMLREKKISRSSALKVLDHAMIGPEGTDNCHKFVDILGLRTIFPL
Stickleback (Gasterosteus aculeatus) LAILLQNNDNTRELLGEMDGIDVLLQQLSVFKRHNPSTAEEQEMMENLFDALCSCLMLPANRDRFLRGEGLQLMNLMLREKKMSRTSSMKVLDHGMIGPEGADNCHKFVDILGLRTIFPL
Gorilla (Gorilla gorilla)            LAILLQDNDENRELLGELDGIDVLLQQLSVFKRHNPSTAEEQEMMENLFDSLCSCLMLSSNRERFLKGEGLQLMNLMLREKKISRSSALKVLDHAMIGPEGTDNCHKFVDILGLRTIFPL
Human (Homo sapiens)                 LAILLQDNDENRELLGELDGIDVLLQQLSVFKRHNPSTAEEQEMMENLFDSLCSCLMLSSNRERFLKGEGLQLMNLMLREKKISRSSALKVLDHAMIGPEGTDNCHKFVDILGLRTIFPL
Elephant (Loxodonta africana)        LAILLQDNDENRELLGELDGIDVLLQQLSVFKRHNPSTAEEQEMMENLFDSLCSCLMLSSNRERFLKGEGLQLMNLMLREKKISRSSALKVLDHAMIGPEGTDNCHKFVDILGLRTIFPL
Opossum (Monodelphis domestica)      LAILLQDNDDNRELLGELDGIDVLLQQLSVFKRHNPGTTEEQEMMENLFDSLCSCLMLSSNRERFLKGEGLQLMNLMLREKKISRSSALKVLDHAMIGPEGTDNCHKFVDILGLRTIFPL
Mouse (Mus musculus)                 LAILLQDNDENRELLGELDGIDVLLQQLSVFKRHNPSTAEEQEMMENLFDALCSCLMLSSNRERFLKGEGLQLMNLMLREKKVSRSSALKVLDHAMIGPEGTDNCHKFVDILGLRTIFPL
Microbat (Myotis lucifugus)          LAILLQDNDENRELLGELDGIDVLLQQLSVFKRHNPSTAEEQEMMENLFDSLCSCLMLSSNRERFLKGEGLQLMNLMLREKKISRSSALKVLDHAMIGPEGTDNCHKFVDILGLRTIFPL
Pika (Ochotona princeps)             LAILLQDNDENRELLGELDGIDVLLQQLSVFKRHNPSTAEEQEMMENLFDSLCSCLMLSSNRERFLKGEGLQLMNLMLREKKISRSSALKVLDHAMIGPEGTDNCHKFVDILGLRTIFPL
Rabbit (Oryctolagus cuniculus)       LAILLQDNDENRELLGELDGIDVLLQQLSVFKRHNPSTAEEQEMMENLFDSLCSCLMLSSNRERFLKGEGLQLMNLMLREKKISRSSALKVLDHAMIGPEGTDNCHKFVDILGLRTIFPL
Medaka (Oryzias latipes)             LAILLQNNDNTRELLGEMDGIDVLLQQLSVFKRHNPNTAEEQEMMENLFDALCSCLMLASNRERFLKGEGLQLMNLMLREKKMSRTSALKVLDHAMIGPEGLDNCHKFVDILGLRTIFPL
Chimpanzee (Pan troglodytes)         LAILLQDNDENRELLGELDGIDVLLQQLSVFKRHNPSTAEEQEMMENLFDSLCSCLMLSSNRERFLKGEGLQLMNLMLREKKISRSSALKVLDHAMIGPEGTDNCHKFVDILGLRTIFPL
Orangutan (Pongo pygmaeus)           LAILLQDNDENRELLGELDGIDVLLQQLSVFKRHNPSTAEEQEMMENLFDSLCSCLMLSSNRERFLKGEGLQLMNLMLREKKISRSSALKVLDHAMIGPEGTDNCHKFVDILGLRTIFPL
Rat (Rattus norvegicus)              LAILLQDNDENRELLGELDGIDVLLQQLSVFKRHNPSTAEEQEMMENLFDALCSCLMLSSNRERFLKGEGLQLMNLMLREKKISRSSALKVLDHAMIGPEGADNCHKFVDILGLRTIFPL
Zebra Finch (Taeniopygia guttata)    LAILLQNNDDNRELLGELDGIDVLLQQLSVFKRHNPSTAEEQEMMENLFDSLCSCLMLSSNRDRFLKGEGLQLMNLMLREKKISRSSALKVLDHAMIGPEGTDNCHKFVDILGLRTIFPL
Fugu (Takifugu rubripes)             LAILLQNNDNTRELLGEMDGIDVLLQQLSVFKRHNPSTAEEQEMMENLFDALCSCLMLSANRERFLRGEGLQLMNLMLREKKISRTSALKVLDHAMIGSEGADNCHKFVDILGLRTIFPL
Tetraodon (Tetraodon nigroviridis)   LAILLQNNDSTRELLGEMDGIDVLLQQLSVFKRHNPSTAEEQEMMENLFDALCSCLMLPANRERFLRGEGLQLMNLMLSAQSRSADSAPDLIDFSI-WPAG------------WVLYLPI
Dolphin (Tursiops truncatus)         LAILLQDNDENRELLGELDGIDVLLQQLSVFKRHNPSTAEEQEMMENLFDSLCSCLMLSSNRERFLKGEGLQLMNLMLREKKISRSSALKVLDHAMIGPEGTDNCHKFVDILGLRTIFPL
Xenopus tropicalis                   LAILLQNNDDTRELLGELDGIDVLLQQLSVFKRHDPGTAEEQEMMENLFDALCSCLMLSSNRDRFLKGEGLQLMNLMLREKKMSRCSALRVLDHAMIGPEGTDNCHKFVDILGLRTIFPL
                                     *:**:* .:. * *:*. :*:*:*****:.:***:*   ** * *:***:.***.*:   ** :** ***********  :. *  .:  ::*..:    *                :*:
```

```
Anole Lizard (Anolis carolinensis)   FMKSPKKIKKV-----GTSEKEHEEHVCSILASLLRNLRGQQRTRLLNKFTENDSEKVDRLMELYFKYLDAMQAADKKIEGEKHDMVRRGEIIDDDME-DEFYLRRLDAGLFILQLICYI
Cow (Bos taurus)                     FMKSPRKIKKV-----GTTEKEHEEHVCSILASLLRNLRGQQRTRLLNKFTENDSEKVDRLMELHFKYLDAVQVADKKIEGEKHDMVRRGEIIDNDIE-DEFYLRRLDAGLFVLQHICYI
Marmoset (Callithrix jacchus)        FMKSPRKIKKV-----GTTEKEHEEHVCSILASLLRNLRGQQRTRLLNKFTENDSEKVDRLMELHFKYLGAMQVADKKIEGEKHDMVRRGEIIDSDIE-EEFYLRRLDAGLFVLQHICYI
Dog (Canis familiaris)               FMKSPRKIKKV-----GTTEKEHEEHVCSILASLLRNLRGQQRTRLLNKFTENDSEKVDRLMELHFKYLDAMQVADKKIEGEKHDMVRRGEIIDNDTE-DEFYLRRLDAGLFVLQHICYI
Guinea Pig (Cavia porcellus)         FMKSPRKIKKV-----GTTEKEHEEHVCSILASLLRNLRGQQRTRLLNKFTENDSEKVDRLMELHFKYLGAVQVADKKIEGEKHDIVRRGEIIDNDLE-DEFYLRRLDAGLFVLQHICYI
Seasquirt (Ciona intestinalis)       FMKPPKKNKKV-----GSSRKDHEEHVISIISSMLQNLSGSHRQRLIAKFTENDFAKVDRLMEFHFKYYERVQRVDNRLDAERTRLLVEGED-EEEIE-EDFYLRRLDAGLFTLQQTDYV
Pacific Seasquirt (Ciona savignyi)   FMKPPKKNKKI-----GSSRKDHEEHSVSIISSMLQNLSGSQRQRLVAKFTENDFAKVDRLMELHFKYYDRVQRVDNRLEAERTRLIAEGED-EEDIE-DDFYLRRLDAGLFTLQQTDYI
Zebrafish (Danio rerio)              FMKTPKKMKKV-----GISDKQHEEHVCSIIASMLRNLKGQQRSRLLSKFTENDCEKVDRLMELHFKYLEAVQLADKRIEGEKHDMVRRGEILDDAMD-DEFYLRRLDAGLFVLQLLCYI
Fruitfly (Drosophila melanogaster)   FMKTPKRNKQR-----LISADEHEEHVTSVIASMLRNCKGTHRQRMLAKFTENDHEKVDRLLELHLKYLAKVEAIDKEIDQQAKDPT----I-DEDEEAENNYIKRLTGGLFTLQRIDYI
Horse (Equus caballus)               FMKSPRKIKKV-----GTTEKEHEEHVCSILASLLRNLRGQQRTRLLNKFTENDSEKVDRLMELHFKYLDAMQVADKKIEGEKHDMVRRGEIIDNDIE-DEFYLRRLDAGLFVLQHICYI
Chicken (Gallus gallus)              FMKSPKKIKKV-----GTTEKEHEEHVCSILASLLRNLRGQQRTRLLNKFTENDSEKVDRLMELYFKYLDAMQAADKKIDGEKHDMVRRGEIIDDDME-DEFYLRRLDAGLFVLQLISYI
Stickleback (Gasterosteus aculeatus) FMKTPKKMKKT-----GASEKEHEEHVCSIIASMLRNLKSQQRSRLLNKFTENDCEKVDRLMEMHFKYLEAVQQADKRIEGEKHEMVRRGEILDDGME-DEFYLRRLDAGLFVLQLICYI
Gorilla (Gorilla gorilla)            FMKSPRKIKKV-----GTTEKEHEEHVCSILASLLRNLRGQQRTRLLNKFTENDSEKVDRLMELHFKYLGAMQVADKKIEGEKHDMVRRGEIIDNDTE-EEFYLRRLDAGLFVLQHICYI
Human (Homo sapiens)                 FMKSPRKIKKV-----GTTEKEHEEHVCSILASLLRNLRGQQRTRLLNKFTENDSEKVDRLMELHFKYLGAMQVADKKIEGEKHDMVRRGEIIDNDTE-EEFYLRRLDAGLFVLQHICYI
Elephant (Loxodonta africana)        FMKSPRKIKKV-----GTTEKEHEEHVCSILASLLRNLRGQQRTRLLNKFTENDSEKVDRLMELHFKYLDAMQVADKKIEGEKHDMVRRGEIIDNDIE-DEFYLRRLDAGLFVLQHICYI
Opossum (Monodelphis domestica)      FMKSPKKIKKV-----GTTEKEHEEHVCSILASLLRNLRGQQRTRLLNKFTENDSEKVDRLMELHFKYLDAMQVADKKIEGEKHDMVRRGEIIDDDIE-DEFYLRRLDAGLFVLQHICYI
Mouse (Mus musculus)                 FMKSPRKIKKV-----GTTEKEHEEHVCSILASLLRNLRGQQRTRLLNKFTENDSEKVDRLMELHFKYLSAMQVADKKIEGEKHDIVRRGEIIDNDME-DEFYLRRLDAGLFILQHICYI
Microbat (Myotis lucifugus)          FMKSPKKIKKV-----GTTEKEHEEHVCSILASLLRNLRGQQRTRLLNKFTENDSEKVDRLMELHFKYLDAMQVADKKIEGEKH-----------------------------------
Pika (Ochotona princeps)             FMKSPRKIKKV-----GTTEKEHEEHVCSILASLLRNLRGQQRTRLLNKFTENDSEKVDRLMELHFKYLGAVQVADKKIEGEKHDMVRRGEIIDNDVD-DELYLRRLDAGLFVLQHICYI
Rabbit (Oryctolagus cuniculus)       FMKSPRKIKKV-----GTTEKEHEEHICSILASLLRNLRGQQRTRLLNKFTENDSEKVDRLMELHFKYLGAMQVADKKIEGEKHDMVRRGEIIDNDMD-DEFYLRRLDAGLFVLQHICYI
Medaka (Oryzias latipes)             FMKTPKKMKKA-----GVPEKEHEEHVCSIIASMLRNLKSQQRTRLLNKFTENDCEKVDRLMELHFKYLEAVQQADKRIEGEKHEMVQRGEILDDSME-DEFYFRRLDAGLFVLQLICYI
Chimpanzee (Pan troglodytes)         FMKSPRKIKKV-----GTTEKEHEEHVCSILASLLRNLRGQQRTRLLNKFTENDSEKVDRLMELHFKYLGAMQVADKKIEGEKHDMVRRGEIIDNDTE-EEFYLRRLDAGLFVLQHICYI
Orangutan (Pongo pygmaeus)           FMKSPRKIKKV-----GTTEKEHEEHVCSILASLLRNLRGQQRTRLLNKFTENDSEKVDRLMELHFKYLGAMQVADKKIEGEKHDMVRRGEIIDSDTE-EEFYLRRLDAGLFVLQHICYI
Rat (Rattus norvegicus)              FMKSPRKIKKV-----GTTEKEHEEHVCSILASLLRNLRGQQRTRLLNKFTENDSEKVDRLMELHFKYLGAMQVADKKIEGEKHDIVRRGEIIDNDME-DEFYLRRLDAGLFILQHICYI
Zebra Finch (Taeniopygia guttata)    FMKSPKKIKKV-----GTTEKEHEEHVCSILASLLRNLRGQQRTRLLNKFTENDSEKVDRLMELYFKYLDAMQVADKKIDGEKHDMVRRGEIIDDDME-DEFYLRRLDAGLFVLQLICYI
Fugu (Takifugu rubripes)             FMKTPKKMKKT-----GTSEKEHEEHVCSIMASMLRNLKSQQRTRLHNKFTENDCEKVDRLMELYFKYLEGVQLADKRIEGEKHEMVRRGEILDDDME-DEFYLRRLDAGLFVLQLICYI
Tetraodon (Tetraodon nigroviridis)   IMIYPNGIRKCKIKVYDPLYSLFSEHVCSIIASMLRNLKSQQRTRLHNKFTENDCEKVDRLMELYFKYLEGVQQADKRIEGEKHEMVRRGEILDDDME-DEFYLRRLDAGLFVLQLICYI
Dolphin (Tursiops truncatus)         FMKSPRKIKKV-----GTTEKEHEEHVCSILASLLRNLRGQQRTRLLNKFTENDSEKVDRLMELHFKYLDAMQVADKKIEGEKHDMVRRGEIIDNDIE-DEFYLRRLDAGLFVLQHICYI
Xenopus tropicalis                   FMKSPKKAKKT-----GVSEKEHEEHVCSILASLLRNLRGQQRTRLLSKFTESDCEKVDRLMELYFKYLEAVRVADKKIEGEKHDMVRRGEILDDDVE-EEFYLRRLDAGLFLLQLLCYV
                                     :*  *.  ::          . ..**  *:::*:*:*  . :* *:  ****.*  *****:*:::**   :.  *:.:: :
```

Anole Lizard (*Anolis carolinensis*)	MAEICNASIPQIRQRVHQILNMRGSSIKIVRHIIKEYAENIGDGKNPEFRESEQKRIVELLDNF
Cow (*Bos taurus*)	MAEICNANVPQIRQRVHQILNMRGSSIKIVRHIIKEYAENIGDGRSPEFRESEQKRILALLENF
Marmoset (*Callithrix jacchus*)	MAEICNANVPQIRQRVHQILNMRGSSIKIVRHIIKEYAENIGDGRSPEFRENEQKRILGLLENF
Dog (*Canis familiaris*)	MAEICNANVPQIRQRVHQILNMRGSSIKIVRHIIKEYAENIGDGRSPEFRENEQKRILGLLENF
Guinea Pig (*Cavia porcellus*)	MAEICNANVPQIRQRVHQILNMRGSSIKIVRHIIKEYAENIGDGRSPEFRDTEQKRILGLLENF
Seasquirt *(Ciona intestinalis)*	LVDICASGSSSIKQRVLQILNLRSGSVKSIRTIIREYVGNLGDNTG-SVSGVDKQRILGLADKF
Pacific Seasquirt *(Ciona savignyi)*	LVDICASGSPSIKQRVLQILNLRSGSVKSIRTIIREFVGNLGDGDN--VSGVDRQRILGLADKF
Zebrafish (*Danio rerio*)	MVEICAAGVPQLQQRVHQILNLRGGSVKVVRHIM-----------------------------R
Fruitfly (*Drosophila melanogaster*)	LLEVS-ATGDTVKQRVLQILNLRGASMKTIRSIMREYAGNLGDGDT-DWREQEQNHILSLVDRF
Horse (*Equus caballus*)	MAEICNANVPQIRQRVHQILNMRGSSIKIVRHIIKEYAENIGDGRSAEFRENEQKRILGLLENF
Chicken (*Gallus gallus*)	MAEICNANVPQIRQRVHQILNMRGSSIKIVRHILKEYAENIGDGKNQEFRESEQKRIMDLLENF
Stickleback (Gasterosteus aculeatus)	MVEISNSGISQLQQRVQQILNLRGGSVKVVRHIMREYAESIGDGKTDEFKEAERKRIMDLVENF
Gorilla (*Gorilla gorilla*)	MAEICNANVPQIRQRVHQILNMRGSSIKIVRHIIKEYAENIGDGRSPEFRENEQKRILGLLENF
Human (*Homo sapiens*)	MAEICNANVPQIRQRVHQILNMRGSSIKIVRHIIKEYAENIGDGRSPEFRENEQKRILGLLENF
Elephant (*Loxodonta africana*)	MAEICNANVPQIRQRVHQILNMRGSSSKVVRHIIKEYAENIGDGRSPEFRENEQKRILGLLENF
Opossum (*Monodelphis domestica*)	MAEICNASVPQIRQRVHQILNMRGSSIKIVRHIIKEYAENIGDGKNQEFRENEQKRILELLENF
Mouse (*Mus musculus*)	MAEICNANVPQIRQRVHQILNMRGSSIKIVRHIIKEYAENIGDGRSPEFRETEQKRILALLENF
Microbat (*Myotis lucifugus*)	--
Pika (*Ochotona princeps*)	MAEICNANVPQIRQRVHQILNMRGSSIKIVRHIIKEYAENIGDGRSPEFRESEQKRILGLLENF
Rabbit (*Oryctolagus cuniculus*)	MAEICNANVPQIRQRVHQILNMRGSSSKIVRHIIK-----------------------------
Medaka (*Oryzias latipes*)	MVEISNSGISQLQQRVHQILNLRGGSVKVVRHIMREYAESLGDGKTEEFKETERKRIVELAENF
Chimpanzee (*Pan troglodytes*)	MAEICNANVPQIRQRVHQILNMRGSSIKIVRHIIKEYAENIGDGRSPEFRENEQKRILGLLENF
Orangutan (*Pongo pygmaeus*)	MAEICNANVPQIRQRVHQILNMRGSSIKIVRHIIKEYAENIGDGRSPEFRENEQKRILGLLENF
Rat (*Rattus norvegicus*)	MAEICNANVPQIRQRVHQILNMRGSSIKIVRHIIKEYAENIGDGRSPEFRETEQKRILGLLENF
Zebra Finch (*Taeniopygia guttata*)	MAEICNANVPQIRQRVHQILNMRGSSIKIVRHILK-----------------------------
Fugu (*Takifugu rubripes*)	MVEINNSGISQLQQRVHQILNLRGGSVKIVRHIMREYIESIGDGKSEEYKEAERKRIMDLVDNF
Tetraodon (*Tetraodon nigroviridis*)	MVEINNSGISQLQQRVHQILNLRGGSAKIVRHIMREYIESIGDGKSEEYKEAERKRVLDLVENF
Dolphin (*Tursiops truncatus*)	MAEICNANVPQIRQRVHQILNMRGSSIKIVRHIIKEYAENIGDGRSPEFRDSEQKRILGLLENF
Xenopus tropicalis	TAEICNNNLPQIRQRVLQILNMRGSSVKIMRHILKEYSESIGDGKSEEFRESEQKRIQELLETL

CLUSTAL FORMAT for T-COFFEE Version_7.71 [http://www.tcoffee.org] [MODE: regular], SCORE=69, Nseq=29, Len=664
